# DEPDC1 facilitated malignant phenotypes and disease progression of liposarcoma by modulating KIF20A

**DOI:** 10.3389/fendo.2025.1591390

**Published:** 2025-06-17

**Authors:** Mingwei Yu, Huishan Zhao, Yujie Sun

**Affiliations:** ^1^ Department of Orthopedics, The Affiliated Yantai Yuhuangding Hospital of Qingdao University, Yantai, China; ^2^ Reproductive Medicine Center, The Affiliated Yantai Yuhuangding Hospital of Qingdao University, Yantai, China

**Keywords:** DEPDC1, KIF20A, liposarcoma, PI3K/AKT/mTOR, malignant phenotype

## Abstract

**Introduction:**

DEP domain containing 1 (DEPDC1) has been well-known as a significant contributor to tumorigenesis and cancer progression. However, its potential oncogenic mechanism in liposarcoma is still unclear.

**Methods:**

In this study, the expression and clinical relevance of DEPDC1 in sarcoma was assessed by employing data from The Cancer Genome Atlas (TCGA) data and conducting Kaplan-Meier online analyses, respectively. Furthermore, the impact of DEPDC1 on cellular functions of liposarcoma cell lines and its underlying mechanisms were studied using the *in vitro* assays.

**Results:**

Here, our findings revealed that the expression levels of DEPDC1 and KIF20A were elevated in liposarcoma compared to the paired adjacent adipose tissues, with their expression positively correlating with the malignancy of liposarcoma. Moreover, patients with high DEPDC1 or KIF20A mRNA levels experienced shorter survival times. *In vitro* assays showed that DEPDC1 overexpression enhanced cell proliferation, migration, and invasion in 93T449 cells, whilst an opposite effect was observed in SW872 cells with DEPDC1 knockdown. Furthermore, potential interacting proteins of DEPDC1 were predicted by STRING, and the DEPDC1-KIF20A interaction was confirmed by co-immunoprecipitation in liposarcoma cells. The deletion of KIF20A partially mitigated the promoting effect of DEPDC1 on the malignant phenotype of liposarcoma cells and the activation of PI3K/AKT/mTOR signaling pathway.

**Conclusions:**

In conclusion, this study suggested that DEPDC1 might interact with KIF20A to promote the occurrence and progression of liposarcoma by activating PI3K/AKT/mTOR signaling pathway.

## 1 Introduction

Liposarcoma are common soft tissue malignancies characterized by multiple histological subtypes, with well-differentiated liposarcoma (WDL) and dedifferentiated liposarcomas (DL) representing the largest subgroups within liposarcoma pathogenesis (1). Surgery, in conjunction with chemotherapy and radiation, constitute the primary treatment for liposarcoma. Despite standard treatments such as radical surgery and systemic chemotherapy, the five-year survival rate for patients with DL remains only 30%, in stark contrast to the 90% survival rate for those with WDL (2). Incomplete resection and the presence of highly malignant liposarcoma continue to adversely impact patient survival. Therefore, further investigation into the pathogenesis of liposarcoma is essential for identifying new diagnostic and therapeutic targets, ultimately enhancing prognosis and improving patients’ survival rate.

DEP domain-containing1 (DEPDC1) is an oncoprotein characterized by the presence of a DEP domain, recognized as a newly identified tumor-related gene first reported in bladder cancer (3). DEPDC1 exhibits overexpression in various malignant tumors, including oral squamous cell carcinoma (4), human osteosarcoma (5, 6), nephroblastoma (7), anaplastic thyroid carcinoma (8), non-small cell lung cancer (9), hepatocellular carcinoma (10, 11), and colorectal cancer (12). The increased expression of the unique RhoGAPs, SDP35/DEDC1A and XPT1/DEPDC1B, is tightly correlated with the metastatic progression of soft-tissue sarcomas, providing compelling evidence for the significant role of aberrant SDP35/DEPDC1A expression as an independent prognostic risk factor (13). Lin et al. (5) had indicated that the suppression of DEPDC1 expression inhibited the proliferation and migration of osteosarcoma cells while promoting cell apoptosis. Furthermore, elevated levels of DEPDC1 expression correlated strongly with reduced survival times and poor prognoses in osteosarcoma patients (6, 14). Recent studies suggested that DEPDC1 protein expression was correlated with poorer TNM stage and recurrence in colorectal cancer (4). DEPDC1 had the capacity to diminish zest in intestinal cancer cells, while the suppression of zest12 (SUZ12) protein expression resulted in a decrease of trimethylation at lysine 27 of histone H3 (H3K27me3) (15). Artemisia was essential oil significantly suppressed DEPDC1 expression in hepatocellular carcinoma, which leading to the downregulation of Wnt/β-catenin signaling and epithelial-mesenchymal transition (10). In hepatocellular carcinoma, DEPDC1 had been validated as a contributor to the reconstruction of the tumor microenvironment, serving as a metabolic gene associated with glycolysis (16). Huang et al. (17) demonstrated that DEPDC1 facilitated the progression of oral squamous cell carcinoma through the WNT/β-catenin signaling pathway. The role of DEPDC1 in promoting cancer development had been increasingly recognized, and it is widely regarded as a putative oncogene. However, currently, the involvement of DEPDC1 in the onset and progression of liposarcoma remains unexplored.

Kinesin family member 20A (KIF20A) is an important member of the kinesin family, exhibiting high expression levels across various cancers. KIF20A microtubules are essential for intracellular transport and play a crucial role in cell division and organization (18, 19). It had been reported that the expression of KIF20A was significantly elevated in several malignancies, including lung adenocarcinoma (20), non-small cell lung cancer (21), prostate cancer (22), fibrosarcoma (23), and colorectal cancer (24, 25). KIF20A served as a pivotal hub gene within the biological regulatory network of soft tissue sarcoma (STS) and was significantly correlated with the prognosis of patients (26). Nevertheless, the precise role and potential mechanisms by which KIF20A influenced the growth and metastasis of liposarcoma remained elusive.

In this study, we would explore the clinical significance and functional implications of DEPDC1 in liposarcoma. We measured the expression levels of DEPDC1 and KIF20A in clinical liposarcoma tissues at mRNA and protein levels. Furthermore, we investigated the mechanisms by which DEPDC1 influenced cell function. We conducted the first investigation of the underlying molecular mechanisms of DEPDC1 dysregulation on the malignant phenotype of liposarcoma cells, which provided new insight into exploring the pathogenesis ofliposarcoma.

## 2 Materials and methods

### 2.1 Study population

26 patients diagnosed as liposarcoma in Yantai Yuhuangding Hospital, from 2021 to 2024, were enrolled in this study. Ethical approval for this study was granted by the Medical Ethics Committee of Yantai Yuhuangding Hospital. The clinicopathological features of the enrolled patients were listed in **Table 1**.

**Table 1:** Clinicopathological features of the enrolled patients.

Variables	Well-dedifferentiated liposarcoma (n = 15)	Well-dedifferentiated liposarcoma (n = 11)	*p* value
Age (years)	55 ± 15.48	55.09 ± 8.65	0.98^a^
Sex_man	10	6	0.69^b^
Sex_woman	5	5	
Surgical quality	Negative margins at the tumor resection boundary	Negative margins at the tumor resection boundary	NA
Neo(adjuvant) chemo/radiotherapy prior to surgery	No	No	NA

a Student t-test.

b fisher’s exact test; NA: not available.

Data presented as mean ± SD. *P* < 0.05was considered statistically significant.

### 2.2 Bioinformatics analysis

Two expression profiles (GSE30929 and GSE159659) were obtained from the Gene Expression Omnibus (GEO) database, which facilitated the detection of differential DEPDC1 and KIF20A expression in liposarcoma with different degrees of lesions. Also, we explored the expression levels of DEPDC1 and KIF20A and their correlation with clinical prognosis based on TCGA-SARC database (The Cancer Genome Atlas) using GEPIA online tool (http://gepia.cancer-pku.cn/index.html). Kaplan-Meier (KM) (https://kmplot.com/analysis/) online tool was used to further identify the correlation between DEPDC1 or KIF20A expression and patient prognosis.

### 2.3 Cell culture and cell transfection

Human liposarcoma cells SW872 and 93T449 were purchased from American Type Culture Collection (ATCC, USA). SW872 cells were cultured in L15 medium (Zhong Qiao Xin Zhou Biotechnology, China), and 93T449 cells were incubated in 1640 medium (Shandong Sparkjade Biotechnology Co., Ltd, China), supplemented with 1% penicillin/streptomycin and10% fetal bovine serum (Biological Industries, Israel) in a cell culture incubator.

The DEPDC1-flag and control vector plasmids were graciously provided by Professor Toyomasa Katagiri from The University of Tokyo, Tokyo, Japan. The siRNAs targeting DEPDC1 and the control siRNA, siRNAs targeting KIF20A and the control siRNA, were chemically synthesized by Shanghai GeneChem (Shanghai, China) and OBIO TECHNOLOGY (Shanghai, China), respectively. One microgram plasmids were transfected into cells using the Lipomaster 3000 Transfection Reagent (VazymeBiotech Co., Ltd., China), in accordance with the manufacturer’s guidelines.

### 2.4 Immunohistochemistry staining

5 well-differentiated liposarcoma (WDL) and 5 differentiated liposarcoma (DL) tissues were sectioned at a thickness of 4µm. The tissue sections were grilled in an 80°C oven for 1 h. Subsequently, slides were dewaxed and rehydrated undergoing xylene and a graded ethanol series. After exposing the sections in antigen retrieval at 100°C for 20 min, incubated them with 3% H_2_O_2_ for 10 min in a wet box. Blocking the sections using 3% bovine serum albumin (BSA, Solarbio) at room temperature for 1 h, the slides were immune stained withanti-DEPDC1 (Abcam, Cambridge, MA, USA) in a humidified box overnight at 4°C. Following three times washing, the sections were incubated with the secondary antibody anti-rabbit for 30 minutes at room temperature. Subsequently, the slides were stained with 3,3′-diaminobenzidine (DAB) and counterstained with hematoxylin according to the manufacturer’s protocol. Following routine dehydration using a gradient ethanol and xylene series, cover slips were mounted onto glass slides. Immunohistochemical images were captured using a Leica Camera.

### 2.5 RNA extraction and quantitative RT-PCR

In accordance with the manufacturer’s protocol, total RNA was extracted from clinical tissues samples and cells utilizing Trizol reagent (VazymeBiotech Co., Ltd.). cDNA was synthesized from each 1 µg RNA sample employing the HiScript^®^ II Q Select RTSuperMix for qPCR (R323, Vazyme Biotech Co., Ltd.).The ChamQ Universal SYBR^®^ qPCR Master Mix (Q711, Vazyme Biotech Co., Ltd.) was employed to assess the expression of mRNAs. The reaction conditions consisted of an initial denaturation at 95°C for 30 seconds, followed by 40 cycles of denaturation at 95°C for 10 seconds and annealing/extension at 60°C for 30 seconds. Relative expression levels of genes were quantified using the 2^−ΔΔCt^ method, with GAPDH serving as the reference gene. The primer sequences were detailed in **Table 2**.

**Table 2:** Primer sequences for RT-qPCR.

Genes	Forward primer (5’-3’)	Reverse primer (5’-3’)
DEPDC1	GAAGCAGTGGATTGGCTTTATG	TCTGGATACCTTCGTGGTAGA
KIF20A	ACCTGATCTGAAGCCCTTGC	TGGTGCTGGTACCTATCCGA
GAPDH	CATGTTCGTCATGGGTGTGAA	GGCATGGACTGTGGTCATGAG

### 2.6 Western blot analysis

Whole cell lysates were resolved using 10% SDS-PAGE and subsequently transferred to a nitrocellulose membrane (Millipore, MA). After blocking nonspecific protein interactions with 5% skimmed milk for 1 hour at room temperature, the membranes were incubated with the appropriate primary antibody [DEPDC1 (Abcam, ab197246), KIF20A (Proteintech, 15911-1-AP), AKT1+AKT2+AKT3 (Abcam, EPR16798), p-AKT (Ser473)(ABclonal, AP1208), PI3K p85 (Cell Signaling Technology, #4292), p-PI3K p85 (Tyr458) (Cell Signaling Technology, #4228), mTOR (CST, #2972), p-mTOR (Ser2448) (Cell Signaling Technology, #2971), and GAPDH (Sangon Biotech, D110016)] overnight at 4°C, followed by treatment with peroxidase-conjugated secondary antibodies for 1 hour at room temperature. The labeled protein bands were visualized with the aid of an ECL detection kit and captured using the ChemiScope 6200 Touch (Clinx, Shanghai, China).

### 2.7 *In vitro* cell proliferation assay

Cells (3000cells/200ul/well) were seeded into 96-well plates and cultured over a period of incubation (Days 1, 2, 3, and 4). Cells were treated with 100 μl 10% Cell Counting Kit-8 solution (VazymeBiotech Co., Ltd., China) and incubated in a cell incubator for 1 h following the manufacturer’s instructions. Ultimately, the absorbance was assessed at 450 nm using a spectrophotometer (BioTekInstruments, Inc., Winooski, USA).

### 2.8 *In vitro* cell migration assay

Human liposarcoma cells were plated into 12-well plates and incubated overnight at 37°C to establish a confluent monolayer. An artificial wound was created in the monolayer using a 10 µl pipette tip, followed by three washes with PBS. The cellular activity was traced and recorded at 0 and 24h using an inverted microscope. The wound was subsequently analyzed using Image J software (version 1.62; National Institute of Health, Bethesda, MD, USA).

### 2.9 *In vitro* cell invasion assay

Transwell chambers featuring polycarbonate filter inserts with an 8-μm pore size (6.5 mm diameter) (Corning, USA) were coated with 50 μg of matrigel (VazymeBiotech Co., Ltd., China) in 100 μl per insert and allowed to air-dry. Following rehydration, 20,000 cells in 200 μl were seeded into the inserts containing 5% fetal calf serum, while the bottom wells were filled with 1 ml of medium supplemented with 10% fetal calf serum. After 48 hours, the cells that had invaded through the matrigel were fixed, stained, and quantified.

### 2.10 Oil Red O staining

Cells were seeded into 6-well plates and cultured in a cell incubator. Cells were fixed with 4% paraformaldehyde, and lipid droplets were visualized utilizing a Modified Oil Red O Staining Kit (Beyotime, China) in accordance with the manufacturer’s protocol.

### 2.11 Immunofluorescence staining assay

SW872 and 93T449 cells were seeded into a 24-well plate covered with coverslips. The next day, cells were fixed with 4% paraformaldehyde (w/v) for 30 min at room temperature. Subsequently, cells were washed with PBS containing 0.1% Triton X-100 for 15 min, then were sealed with 3% BSA (dissolved in PBS with 0.1% Triton X-100) for 1 h at room temperature. To distinguish the localization of DEPDC1 and KIF20A, the slides were incubated with primary antibodies from different species at 4°C overnight. Following incubation of the slips using FITC-labeled goat anti-rabbit IgG (H+L) antibody and TRITC-labeled anti-rabbit IgG (H+L) antibody (1:200, for 1 h, DAPI was used for cell nuclear staining. Representative images were captured by a Zeiss fluorescence microscope (Axio Observer 7, German).

### 2.12 Co-immunoprecipitation assay

Aim to find the interacting proteins of DEPDC1, we constructed a protein‐protein interaction (PPI) network based on the STRING website (http://string-db.org/, version 11.0) to visualize the interactions among multiple proteins. The coimmunoprecipitation (CO-IP) assay was performed using a kit (Absin, China). The cells supernatant was bound toanti-DEPDC1 antibody (abcam, ab197246) or normal rabbit IgG (Cell Signaling Technology, #2729) on a rotating shaker at 4°C. The following day, protein complexes were purified after washing with the IP washing buffer. Based on the PPI results, we selected KIF20A as the target protein for further identification.

### 2.13 Statistical analysis

All samples were analyzed in triplicate, and experiments were independently repeated three times. The data were presented as the mean ± standard deviation. The results were analyzed using either a paired or unpaired test to compare two groups, or the one-way analysis of variance (ANOVA) to compare multiple groups. p<0.05 indicated statistically significant.

## 3 Results

### 3.1 Abnormally elevated expression of DEPD1 in human liposarcoma

**Figure 1:**
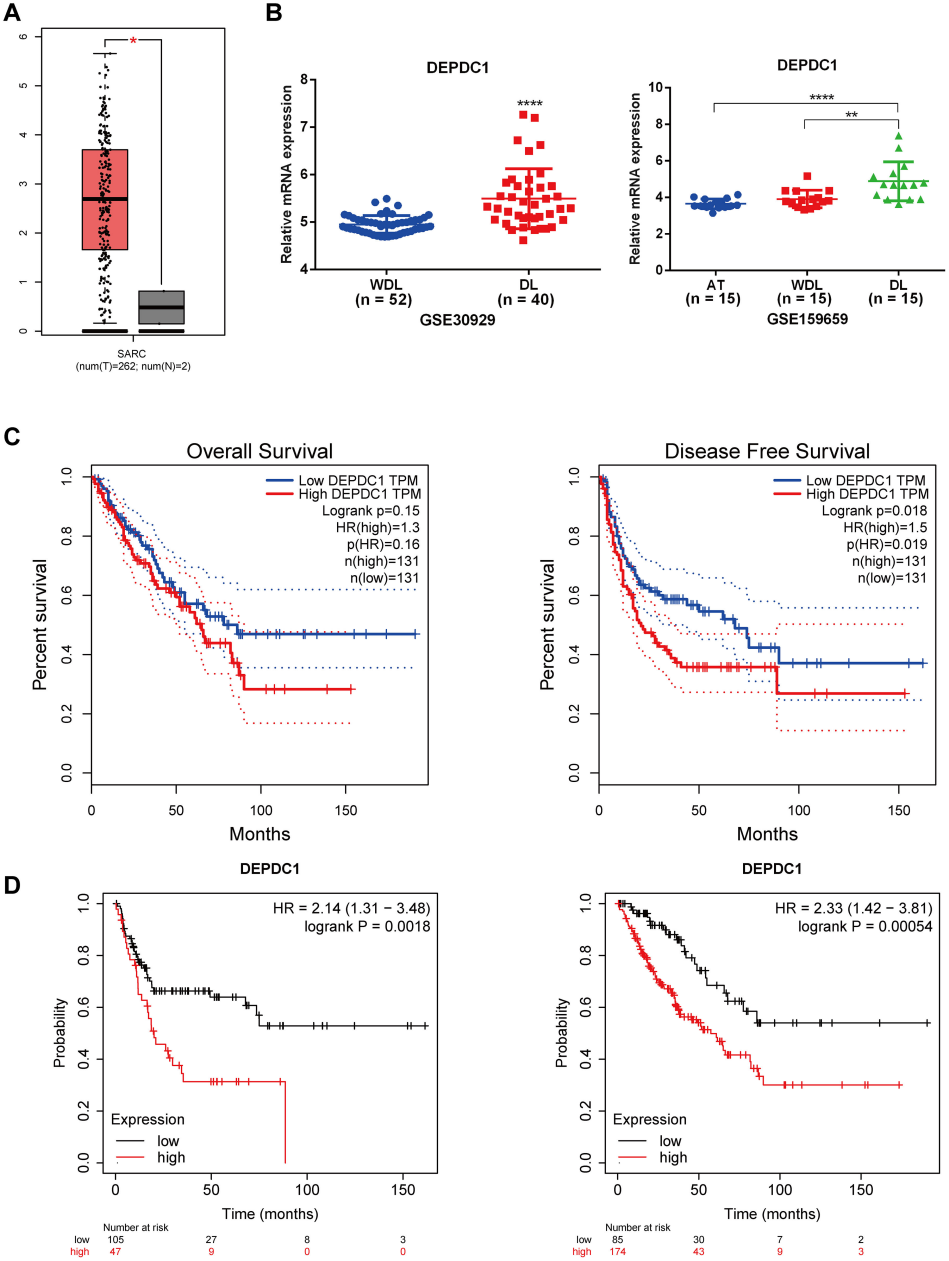
High Expression of DEPDC1 in Dedifferentiated Liposarcoma and its association with poor prognosis of sarcoma patients. **(A)** The mRNA level of DEPDC1 was significantly elevated in sarcoma tissues by analyzing the TCGA_SARC database via searching GEPIA website. **(B)** The mRNA level of DEPDC1 in dedifferentiated liposarcoma and well-differentiated liposarcoma in GEO datasets (GSE30929 and GSE159659). **(C)** The high expression of DEPDC1 was significantly correlated with shorter overall survival (OS) and disease-free survival (DFS) in sarcoma patients. **(D)** Patients with high DEPDC1 expression in sarcoma exhibited shorter OS and DFS via analyzing the Kaplan–Meier (KM) online data. (*p < 0.05; **p < 0.01; ****p < 0.0001).

Since the TCGA database did not distinguish between sarcoma subtypes, we analyzed the expression level of DEPDC1 across sarcomas and its correlation with patients prognosis. As shown in **Figure 1A**, it illustrated that DEPDC1 was aberrantly upregulated in sarcomas using GEPIA online tool (http://gepia.cancer-pku.cn/index.html). Furthermore, we screened and analyzed liposarcoma-related data from the GEO database, and found that DEPDC1 expression was significantly elevated in dedifferentiated liposarcoma (DL) compared to well-dedifferentiated liposarcoma (WDL). Coincidentally, the GSE159659 data revealed that DEPDC1 exhibited the highest expression level and achieved statistical significance in DL, while no significant difference in expression was observed between adipose tissues (AT) and WDL (**Figure 1B**). The GEPIA online tool indicated that DEPDC1 expression was linked to patients’ survival in sarcoma. High DEPDC1 expression was significantly associated with shorter disease-free survival (DFS) in sarcoma patients (**Figure 1C**). Kaplan–Meier (KM) curves further demonstrated that high DEPDC1 expression correlated with reduced overall survival (OS) and DFS in patients with sarcoma (**Figure 1D**). These results demonstrated that DEPDC1 expression was linked to the occurrence and development of liposarcoma and was negatively associated with the survival of sarcoma patients.

### 3.2 The expression of DEPDC1 correlated with the malignancy of liposarcoma

**Figure 2:**
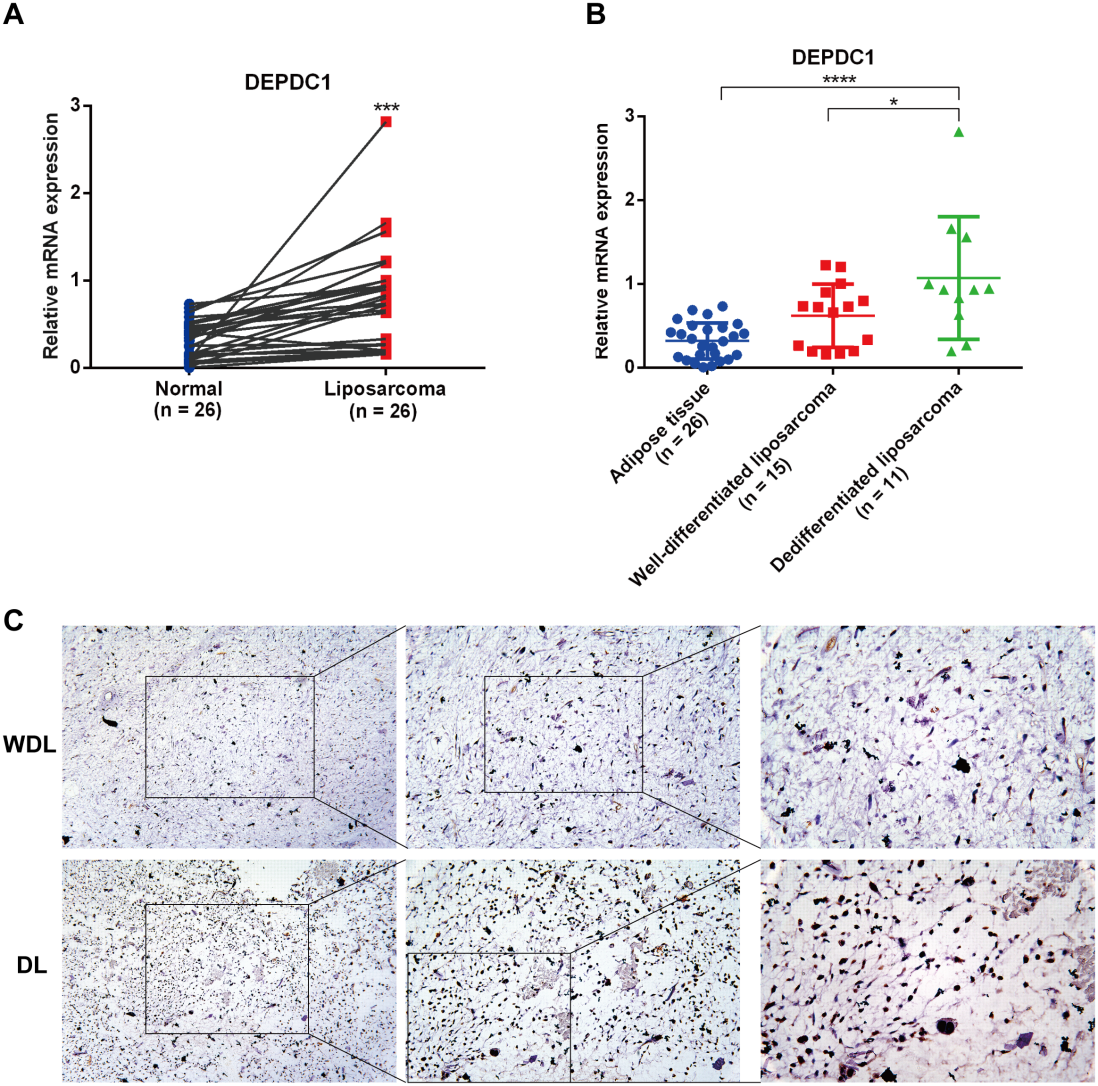
The expression of DEPDC1 correlated with the malignancy of liposarcoma. **(A)** DEPDC1 was significantly upregulated in liposarcoma (n = 26) (paired t-test). **(B)** The expression level of DEPDC1 in AT (n = 26), WDL (n = 15) and DL (n = 11) using qPCR. **(C)** IHC analysis revealed that the protein level of DEPDC1 was markedly higher in DL (n = 5) compared to WDL (n = 5). (*p < 0.05; ***p < 0.001; ****p < 0.0001).

To further identify the expression of DEPDC1 in clinical samples, we collected liposarcoma and adjacent adipose tissue with varying degrees of malignancy and analyzed the expression of DEPDC1 across these different tissues using qPCR and IHC. The qPCR results revealed that the DEPDC1 mRNA level in liposarcoma tissues was significantly higher than in normal adipose tissues from the same patients with liposarcoma (n = 26), as determined by a paired t-test (**Figure 2A**). Furthermore, the abundance of DEPDC1 progressively increased in tissues corresponding to the malignancy of liposarcoma (AT = 26; WDL = 15; DL = 11); however, the expression of DEPDC1 between AT and WDL tissues did not reach a statistical significance (**Figure 2B**). Interestingly, the expression tendency of DEPDC1 in WDL and DL tissues at the protein level (n=5 per group) was consistent with mRNA level (**Figure 1C**). Therefore, the clinical samples confirmed that the expression of DEPDC1 was positively correlated with the malignancy of liposarcoma and might hold prognostic significance for liposarcoma patients.

### 3.3 DEPDC1 promoted the progression of the malignant phenotype in liposarcoma cells

**Figure 3:**
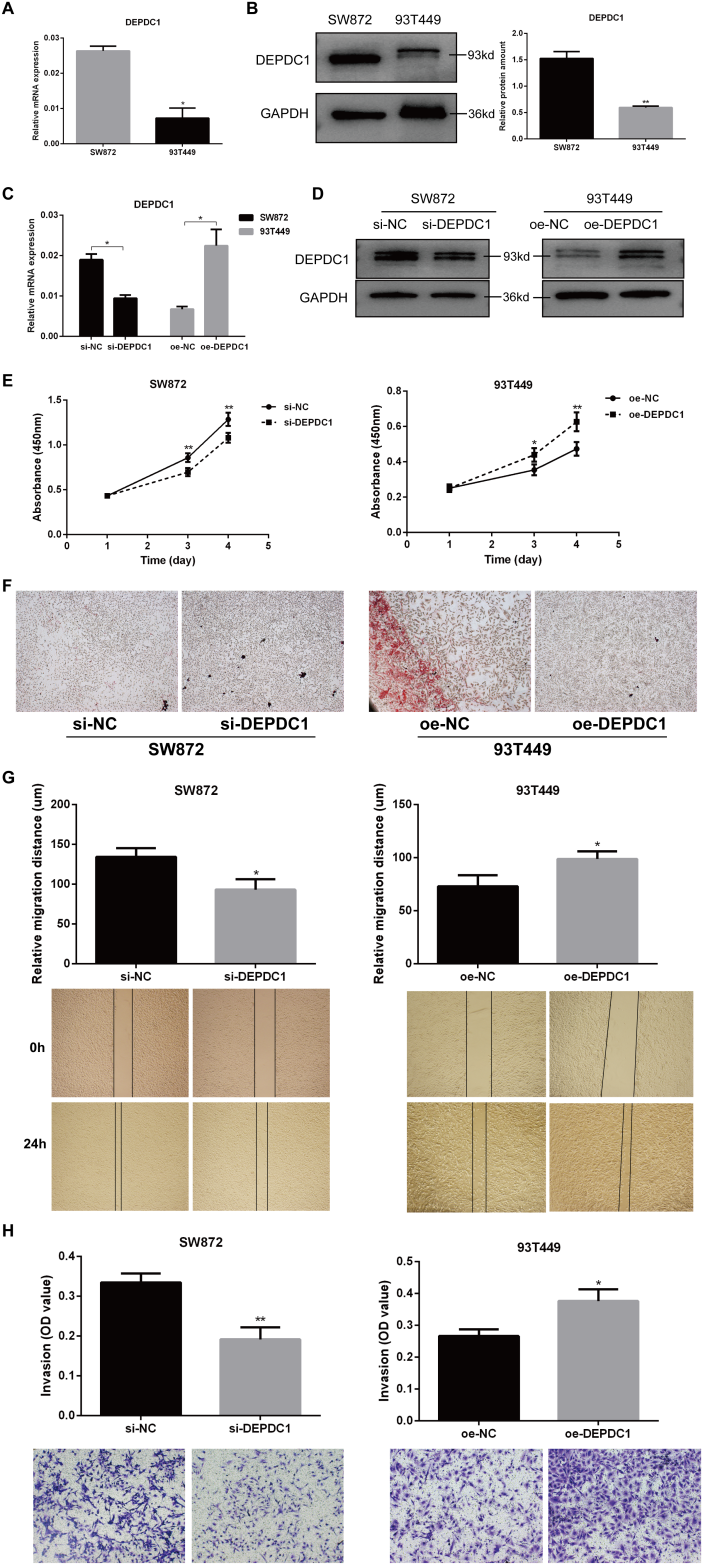
DEPDC1 promoted the progression of the malignant phenotype in liposarcoma cells. **(A)** qPCR results showed the expression levels of DEPDC1 mRNA in SW872 and 93T449 cells. **(B)** Western blot analysis demonstrated that DEPDC1 protein levels were higher in SW872 cells and relatively lower in 93T449 cells. **(C, D)** Stable overexpression of DEPDC1 mRNA and protein in 93T449 cells and silencing in SW872 cells through transfection and selection. **(E)** DEPDC1 knockdown inhibited the proliferation of SW872 cells, while its overexpression promoted the proliferation of 93T449 cells. **(F)** Oil Red O staining showed the lipid accumulation in two liposarcoma cell lines. **(G)** Wound healing analysis of wound closure (cell migration) in liposarcoma cells after the overexpression or knockdown of DEPDC1. **(H)** The effect of DEPDC1 on the invasion of liposarcoma cells. (*p < 0.05; **p < 0.01).

We next evaluated the biological functions of DEPDC1 in liposarcoma by employing overexpression and deletion techniques in the SW872 and 93T449 cell lines. The qPCR results revealed that DEPDC1 expression was low in 93T449 cells and high in SW872 cells (**Figure 3A**). Interestingly, the expression pattern of DEPDC1 at the protein level is consistent with that at the gene level in both cell lines (**Figure 3B**). Consequently, through transfection and selection, DEPDC1 mRNA and protein were stably overexpressed in 93T449 cells and silenced in SW872 cells. DEPDC1 expression was confirmed via qPCR add western blot analysis (**Figures 3C, D**).

The CCK-8 assay revealed that DEPDC1 overexpression enhanced proliferation, while DEPDC1 depletion reduced the proliferation rate in liposarcoma cells. Knockdown of DEPDC1 inhibited proliferation in SW872 cells, whereas its overexpression stimulated proliferation in 93T449 cells (**Figure 3E**). The reduction in lipid accumulation signified a manifestation of adipose tissue damage. Oil Red O staining revealed increased lipid aggregation in the SW872 group with siDEPDC1, while it was diminished in 93T449 cells overexpressing DEPDC1 (**Figure 3F**). Migration and invasion significantly influence the metastasis of liposarcoma cells. The scratch assay demonstrated that the overexpression of DEPDC1 markedly enhanced the migration of 93T449 cells in comparison to the control group, whereas the knockdown resulted in a diminished migration rate in the SW872 cell monolayer, and it presented the representative images of the migration assays for SW872 and 93T449 cells at 0 and 24 h (**Figure 3G**). The invasion assay revealed that the knockdown of DEPDC1 suppressed the invasion of SW872 cells, whereas its overexpression facilitated the invasion of 93T449 cells (**Figure 3H**). In brief, the above results suggested a positive correlation between the expression level of DEPDC1 and the malignant phenotype of liposarcoma cells.

### 3.4 The DEPDC1 protein interacted with KIF20A in liposarcoma cells

**Figure 4:**
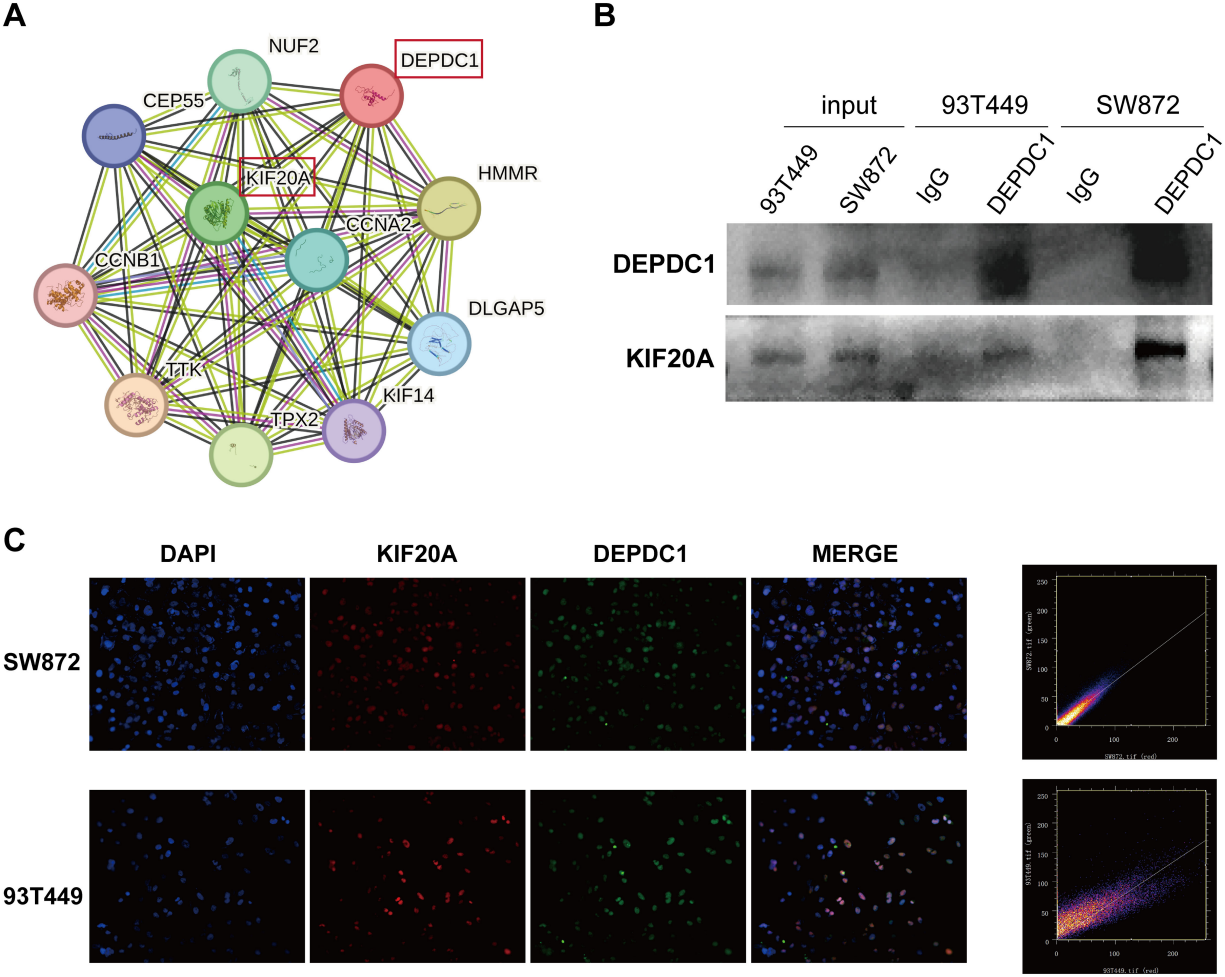
The interaction between DEPDC1 and KIF20A. **(A)** The STRING database revealed the interacting proteins of DEPDC1. **(B)** Verification of the interaction of DEPDC1 and KIF20A in liposarcoma cells by co-immunoprecipitation. **(C)** The co- localization of the DEPDC1 and KIF20A proteins in liposarcoma cells using IF. The Pearson’s correlation coefficient for colocalization was 0.92 in SW872 and 0.83 in 93T449.

To identify the proteins that interacted with DEPDC1, we constructed a protein-protein interaction (PPI) network utilizing the STRING database. As shown in **Figure 4A**, it presented ten proteins closely associated with DEPDC1. A previous study reported that the knockdown of KIF20A suppressed tumor growth in soft tissue sarcoma, both *in vitro* and *in vivo* (27). Therefore, KIF20A was chosen as a candidate target protein. Furthermore, the interaction between DEPDC1 and KIF20A proteins was corroborated by CO-IP experiments (**Figure 4B**). In addition, we validated the co-localization of two proteins. The IF results showed that DEPDC1 and KIF20A co-localized and were expressed within the nucleus of both SW872 and 93T449 cells (**Figure 4C**). Moreover, colocalization is depicted through a scatterplot, illustrating pixel intensities from each channel along the respective axes, using Image J software. Additionally, we calculated the Pearson’s correlation coefficient for colocalization, which yielded a value of 0.92 in SW872 and 0.83 in 93T449. These findings suggested that DEPDC1 mediated malignant phenotype of liposarcoma cells at least partially by interacting with KIF20A.

### 3.5 Expression levels of KIF20A in clinical samples and public databases of liposarcoma

**Figure 5:**
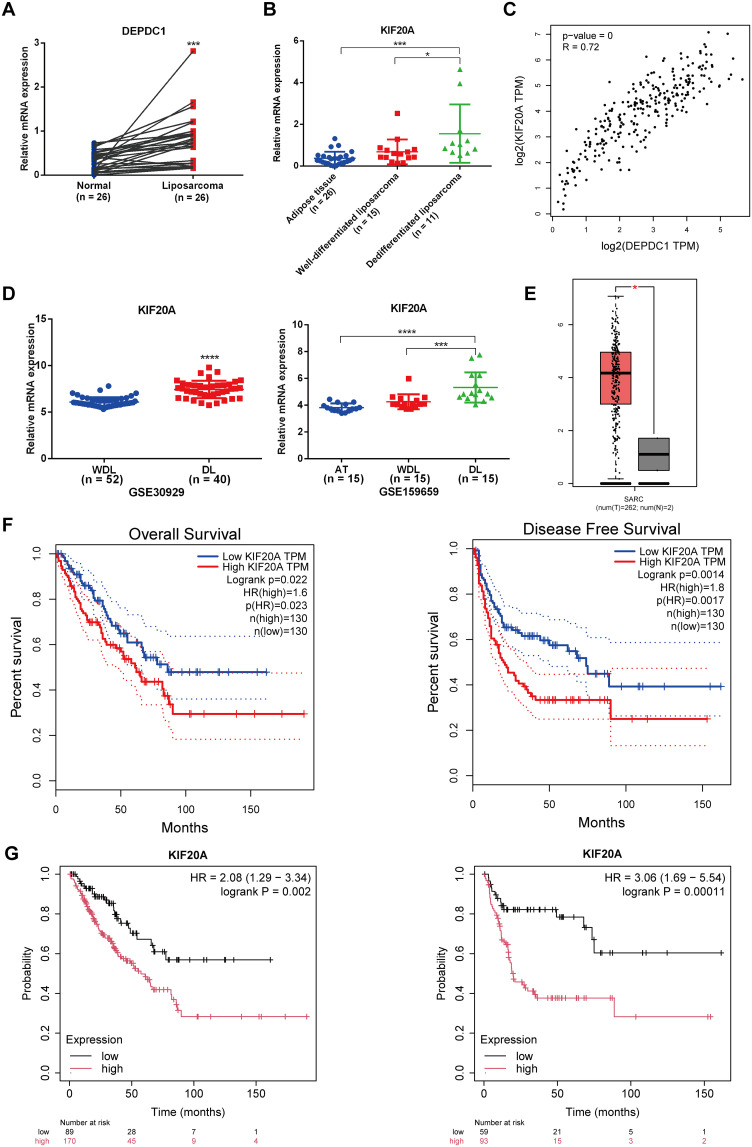
Expression levels of KIF20A in liposarcoma clinical samples and public databases. **(A)** The expression of KIF20A in liposarcoma clinical samples compared to adipose tissue (paired t-test). **(B)** The expression level of KIF20A in AT, WDL and DL using qPCR. **(C)** The positive correlation between the expression of KIF20A and DEPDC1 in sarcomas. **(D)** Analysis of the GEO database (GSE30929 and GSE159659) revealed significantly higher KIF20A mRNA expression in DL compared to WDL. **(E)** The GEPIA website showed abnormal upregulation of KIF20A mRNA levels in sarcomas. **(F)** Patients with high KIF20A expression in sarcomas had shorter OS and DFS times. **(G)** Kaplan–Meier (KM) analysis indicated that high DEPDC1 expression in sarcoma patients was significantly associated with poor prognosis. (*p < 0.05; ***p < 0.001; ****p < 0.0001).

The qPCR results revealed that the KIF20A mRNA level in liposarcoma tissues was significantly higher than in normal adipose tissues from the same patients with liposarcoma (n = 26), as determined by a paired t-test (**Figure 5A**). Moreover, the abundance of KIF20A progressively increased in tissues corresponding to the malignancy of liposarcoma (AT = 26; WDL = 15; DL = 11); however, the expression of KIF20A between AT and WDL tissues did not reach a statistical significance (**Figure 5B**). The GEPIA website indicated a positive correlation between KIF20A and DEPDC1 expression in sarcoma (**Figure 5C**). Through an analysis of the GEO database (GSE30929 and GSE159659), we found that the mRNA levels of KIF20A were significantly more elevated in DL compared to WDL (**Figure 5D**). The GEPIA website revealed that the mRNA levels of KIF20A were aberrantly upregulated in sarcomas (**Figure 5E**). Patients exhibiting high KIF20A expression in sarcomas demonstrated reduced OS and DFS durations (**Figure 5F**). Furthermore, the Kaplan-Meier (KM) online platform indicated that patients with elevated DEPDC1 expression in sarcomas were significantly associated with a poor prognosis (**Figure 5G**).

### 3.6 The knockdown of KIF20A partially mitigated the impact of DEPDC1 overexpression on the malignant phenotype of liposarcoma cells

**Figure 6:**
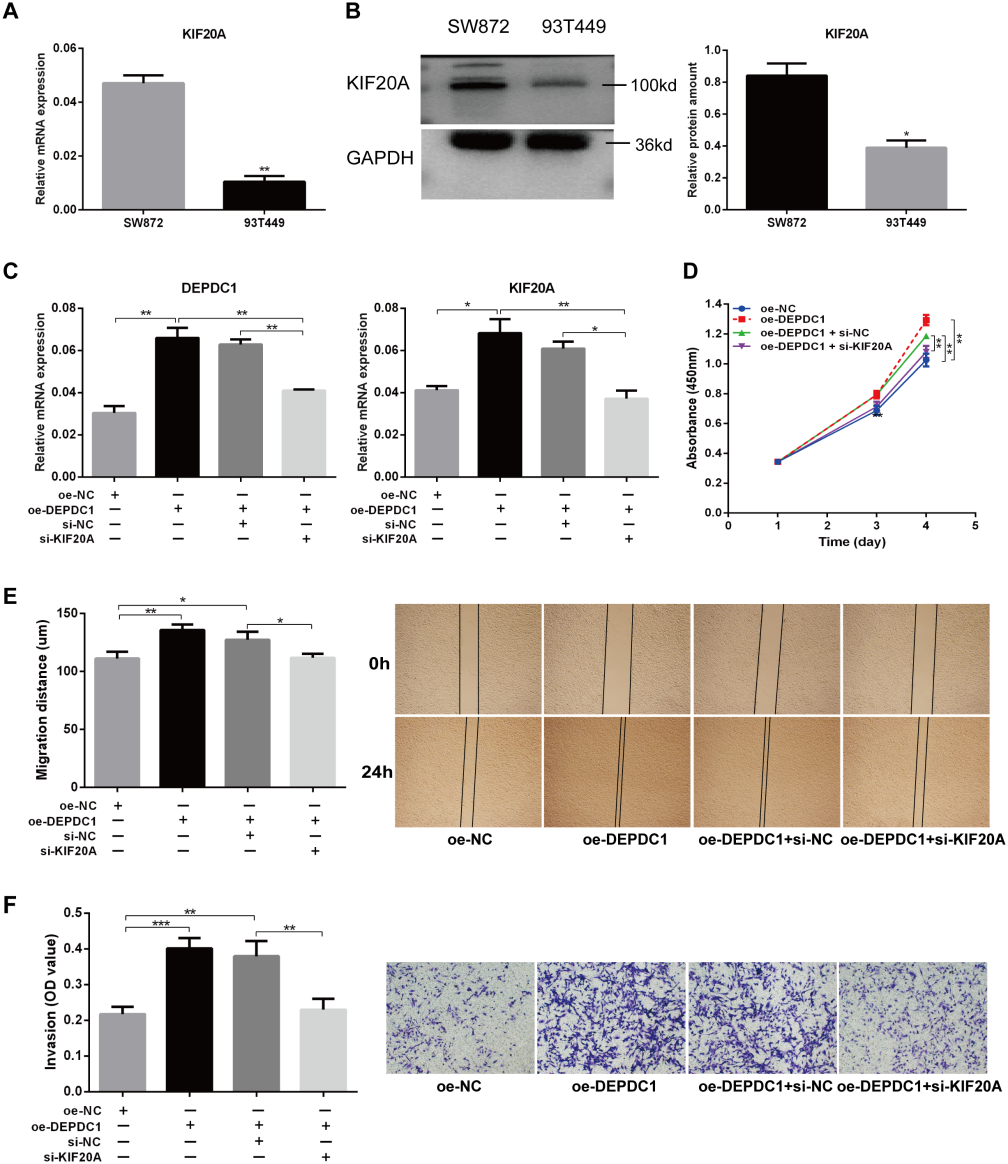
Knockdown of KIF20A partially reversed the effect of DEPDC1 overexpression on the malignant phenotype of liposarcoma cells. **(A, B)** Expression of KIF20A mRNA and protein in two liposarcoma cell lines. **(C)** Validation of DEPDC1 and KIF20A expression after DEPDC1 overexpression and KIF20A knockdown. **(D)** The cell proliferation following DEPDC1 overexpression and KIF20A knockdown using CCK8 assay. **(E)** Scratch assay to assess the impact of DEPDC1 overexpression and KIF20A knockdown on cell migration. **(F)** Transwell assay to evaluate the effect of DEPDC1 overexpression and KIF20A knockdown on cell invasion. (*p < 0.05; **p < 0.01; ***p < 0.001).

The qPCR results demonstrated that KIF20A mRNA expression was elevated in SW872 cells and relatively low in 93T449 cells (**Figure 6A**). Western blot analysis demonstrated that the expression pattern of DEPDC1 at the protein level was consistent with its mRNA expression in both cell lines (**Figure 6B**). To explore whether DEPDC1 promoted the malignant phenotype of liposarcoma cells via regulating KIF20A, DEPDC1 overexpression with knockdown of KIF20A was performed in SW872 cells (**Figure 6C**). The regulation of KIF20A reversed the increased viability and proliferation of SW872 cells caused by the upregulation of DEPDC1 (**Figures 6D, E**). **Figure 6E** presents representative images of the migration assays for liposarcoma cells at 0 and 24 hours. The transwell assay revealed that siKIF20A partially attenuated the invasion ability of liposarcoma cells induced by DEPDC1 overexpression (**Figure 6F**). In brief, these results indicated that DEPDC1 facilitated malignant phenotypes by modulating KIF20A in liposarcoma cells.

### 3.7 The effect of siKIF20A counteracted the activation of the PI3K/AKT/mTOR pathway induced by DEPDC1 overexpression in liposarcoma cells

**Figure 7:**
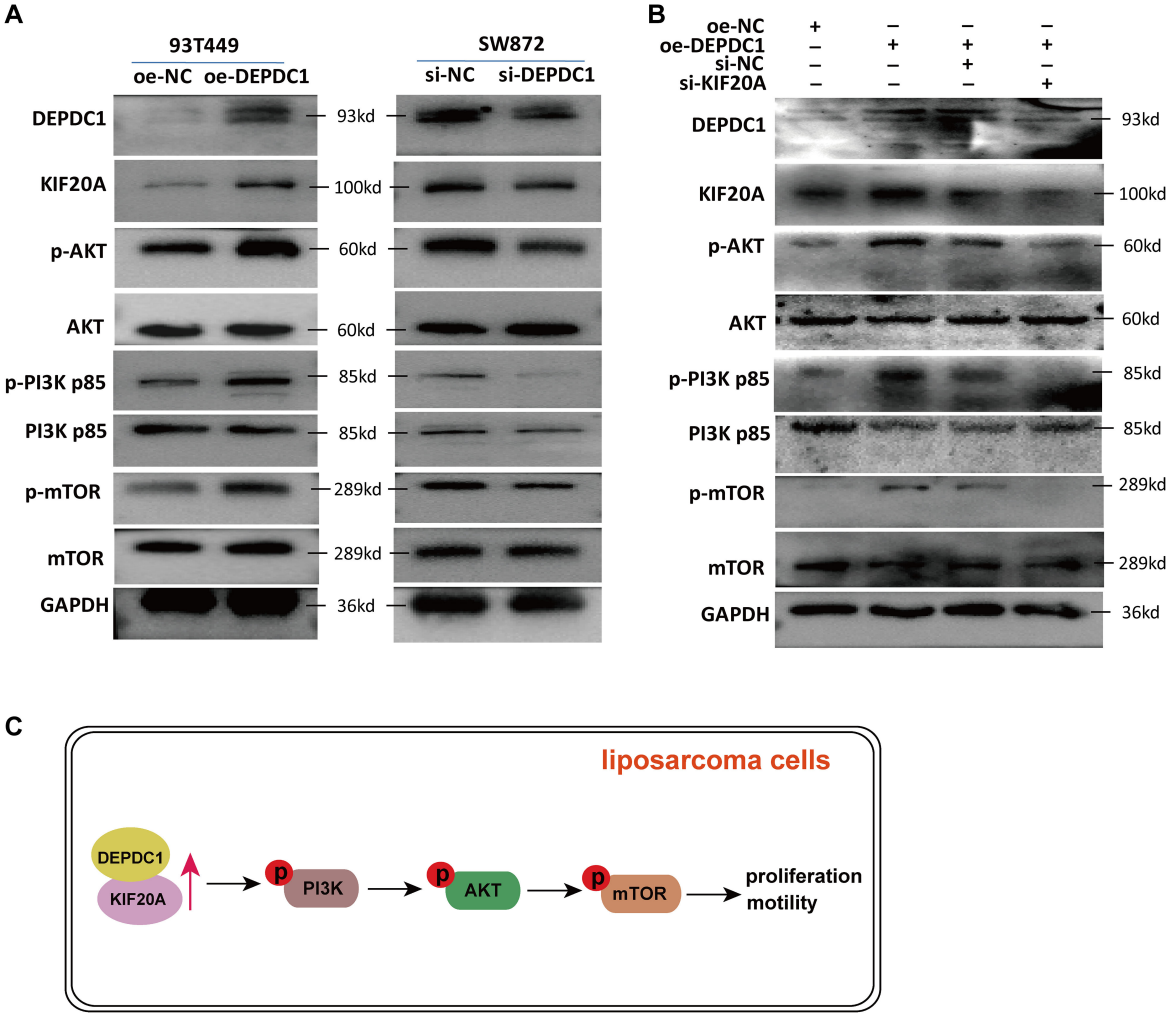
si-KIF20 counteracted the activation of the AKT/PI3K/mTOR pathway induced by DEPDC1 overexpression. **(A)** In 93T449 cells, DEPDC1 overexpression activated the phosphorylation level of PI3K p85 (Tyr458), AKT (Ser473) and mTOR (Ser2448); in SW872 cells, DEPDC1 knockdown inhibited the activation of the AKT/PI3K/mTOR pathway. **(B)** si-KIF20 diminished the activation of the AKT/PI3K/mTOR pathway induced by DEPDC1 overexpression. **(C)** A graphical abstract underlying the mechanim by which DEPDC1 promotes aggressive behaviour thought KIF20A in liposarcoma.

Our previous study revealed that DEPDC1 was exclusively associated with the PI3K/AKT/mTOR signaling pathway in breast cancer, and key genes involved in the activation of this pathway were enriched in patients with elevated DEPDC1 expression (28). The published studies demonstrated elevated activation of the PI3K/AKT/mTOR and MAPK pathways in liposarcoma, as compared to benign adipose tissue (29). Our study found that the overexpression of DEPDC1 triggered the activation of the AKT/PI3K/mTOR signaling pathway in 93T449 cells. In contrast, knockdown of DEPDC1 in SW872 cells inhibited the activation of the AKT/PI3K/mTOR signaling pathway (**Figure 7A**). Furthermore, the PI3K/AKT/mTOR signaling pathway plays a pivotal role in various cellular processes that contribute to the malignant phenotype of liposarcoma. The effect of siKIF20A diminished the activation of the AKT/PI3K/mTOR pathway induced by DEPDC1 overexpression (**Figure 7B**). We proposed that in liposarcoma, the expression of DEPDC1 was positively correlated with the activation of the PI3K/AKT/mTOR pathway. The effect of si-KIF20A antagonized the activating influence of DEPDC1 overexpression on AKT/PI3K/mTOR in liposarcoma cells.

## 4 Discussion

Soft tissue sarcomas arise from adipose tissue, fascia, muscle, fibrous tissue, lymphatic structures, and blood vessels, each exhibiting distinct histological and biological characteristics, as well as varying propensities for local infiltration, hematogenous spread, and lymphatic metastasis. Lung metastases are particularly prevalent, with liposarcoma representing a sarcoma of adipose origin among soft tissue sarcomas. Liposarcoma stands as the most common subtype within this category (30). Soft tissue sarcomas constitute a heterogeneous and biologically diverse group of mesenchymal tumors, characterized by a wide range of clinical behaviors and treatment modalities. Currently, surgical intervention remains an effective approach for managing liposarcoma; however, early diagnosis and precise treatment pose significant challenges. Traditional early screening methods include ultrasound and magnetic resonance imaging, yet biomarkers for liposarcoma have not been integrated into clinical diagnostics and lack the capacity for early, specific detection of the disease.

Currently, the role of DEPDC1 in promoting cancer development has been progressively unveiled, and it is widely regarded as a potential oncogene. Research has demonstrated that DEPDC1 is highly expressed in a majority of tumors, including human osteosarcoma (5, 6), hepatocellular carcinoma (10, 11), nephroblastoma (7), anaplastic thyroid carcinoma (8), colorectal cancer (4), and oral squamous cell carcinoma. Related studies on DEPDC1 in soft tissue sarcoma or osteosarcoma had been published, all of which indicated that DEPDC1 played a significant role in facilitating tumor development (5, 6, 13, 31). However, as a significant entity within soft tissue sarcomas, the biological functions and clinical significance of liposarcoma remain inadequately understood. In this study, we found DEPDC1 expression was linked to the occurrence and development of liposarcoma and was negatively associated with the survival of sarcoma patients. The expression of DEPDC1was correlated with the prognosis and survival time of tumor patients. Amisaki et al. (11) found that compared to normal liver tissue, DEPDC1 was elevated in the carcinoma tissues of patients with hepatocellular carcinoma. Furthermore, the high expression of DEPDC1 in tumor tissues was linked to tumor progression and poor prognosis. Clinical tissue samples from patients with renal cell carcinoma revealed that DEPDC1 was associated with unfavorable prognosis and served as a predictor of renal cell carcinoma metastasis (32). Therefore, DEPDC1 acted as a biomarker for the early detection and prediction of survival in various tumors.

To clarify the precise mechanistic role of DEPDC1 in liposarcoma progression, a bioinformatics analysis was performed, which revealed KIF20A as a downstream target gene of DEPDC1. We have highlighted that KIF20A is upregulated in various cancers and is strongly correlated with poor overall survival. To the best of our knowledge, the role and underlying mechanisms of KIF20A in liposarcoma remain inadequately explored. KIF20A, also referred to as Kinesin Family 20A, has been implicated in the progression of several malignancies, including bladder, prostate, pancreatic ductal adenocarcinoma, and liver cancer. Increased expression of KIF4A was indicative of a poor prognosis and contributes to tumor growth in osteosarcoma. Conversely, the downregulation of KIF4A can suppress colony formation, invasion, migration, and disrupt the cell cycle of osteosarcoma cells (33). Yang et al. (6) demonstrated that the overexpression of KIF4A amplified the effects of DEPDC1 interference, promoting the proliferation, invasion, and migration of U2OS cells, as well as the formation of HUVEC tubes. Furthermore, downregulation of DEPDC1 activated the Hippo signaling pathway, leading to the overexpression of p-LATS1 and p-YAP, which in turn inhibited YAP and suppressed the proliferation of osteosarcoma cells. Indeed, the role of KIF20A in liposarcoma remains unexplored in the current scientific literature. While KIF20A has been implicated in promoting tumorigenesis in other cancers, its specific involvement in liposarcoma requires further investigation. Future research is needed to determine whether KIF20A plays a similar role in liposarcoma, particularly in processes like cell proliferation, migration, and invasion. In our study, differential expression analysis and hub gene identification revealed that KIF20A was differentially expressed between liposarcoma and normal tissues. Furthermore, it played a pivotal role in regulating the network of protein interactions, highlighting its potential significance in liposarcoma pathogenesis. Moreover, we discovered that the DEPDC1 protein interacted with KIF20A and colocalized with it in the nucleus of SW872 and 93T449 cells. Zheng et al. (23) found that KIF20A regulated the proliferation and metastasis of fibrosarcoma through the phosphoinositide 3-kinase (PI3K)-Akt signaling pathway. Also, we found DEPDC1 could activate PI3K/AKT/mTOR signaling pathway. Furthermore, the activation of the PI3K/AKT/mTOR signaling pathway in liposarcoma significantly enhances the rate of cell proliferation and cellular vitality (34, 35). Moreover, the overexpression of DEPDC1, coupled with the simultaneous knockdown of KIF20A in liposarcoma cells, partially inhibited the progression in malignant phenotypes of these cells and the activation of PI3K/AKT/mTOR signaling pathway compared to the control groups. This result suggests that DEPDC1 may influence the malignant phenotype by modulating KIF20A to activate the PI3K/AKT/mTOR signaling pathway in liposarcoma. Certainly, more comprehensive research was required to draw a clear conclusion.

In conclusion, the expression of DEPDC1 and KIF20A was elevated in liposarcoma. The upregulation of DEPDC1 promoted the proliferation, invasion, and migration of liposarcoma cells, which could be reversed by downregulating KIF20A. *In vitro* cell functional assays indicated that DEPDC1 acts as an oncogene in liposarcoma. Furthermore, this study uncovered a novel regulatory mechanism of DEPDC1 in liposarcoma, wherein it enhanced PI3K/AKT/mTOR signaling to exacerbate malignant phenotypes. However, we did not perform the pharmacological inhibition of PI3K or Akt experiments to validate that DEPDC1 induces the proliferation and motility of liposarcoma cells through the PI3K/Akt/mTOR signaling pathway. Further in-depth molecular biology experiments on the mechanisms underlying the increase in DEPDC1 levels in liposarcoma patients should be conducted with animal models. Our findings offered new insights that could enhance the current understanding of liposarcoma pathogenesis.

## Data Availability

The original contributions presented in the study are included in the article/**Supplementary Material**. Further inquiries can be directed to the corresponding authors.
