# Supplementary figures and images for: DEPDC1 facilitated malignant phenotypes and disease progression of liposarcoma by modulating KIF20A
Source: Front Endocrinol (Lausanne). 2025 Jun 17;16:1591390. doi: 10.3389/fendo.2025.1591390 (PMC12208836; doi:10.3389/fendo.2025.1591390)

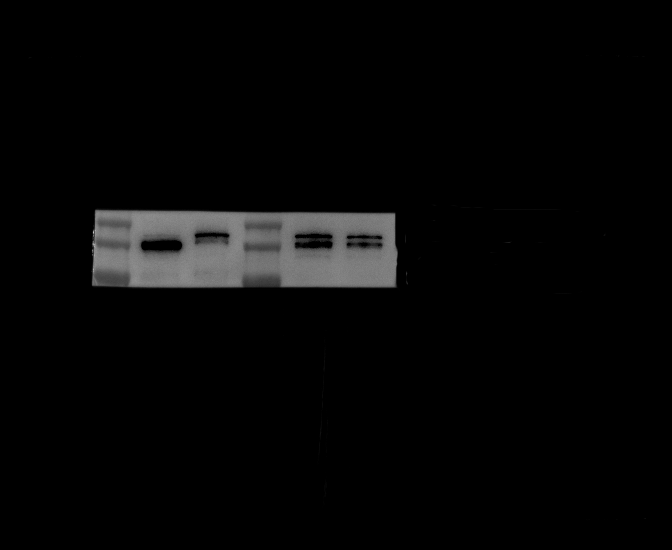

Supplement: Supplementary file 1 [file DataSheet1.zip › original wb/1-DEPDC1.tif]

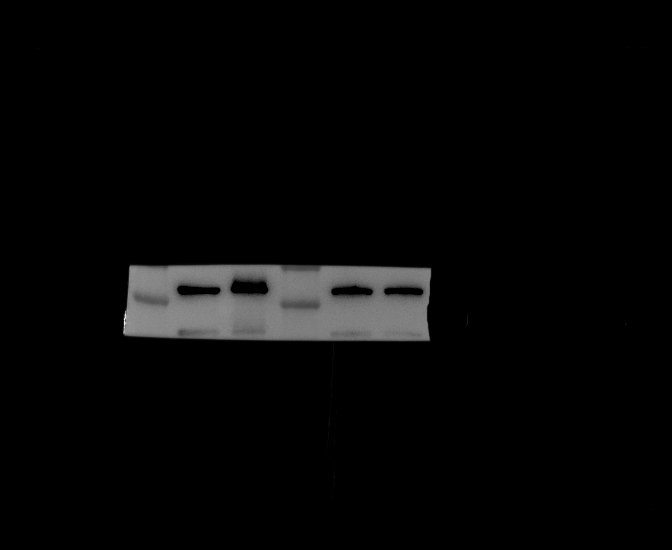

Supplement: Supplementary file 1 [file DataSheet1.zip › original wb/1-GAPDH.tif]

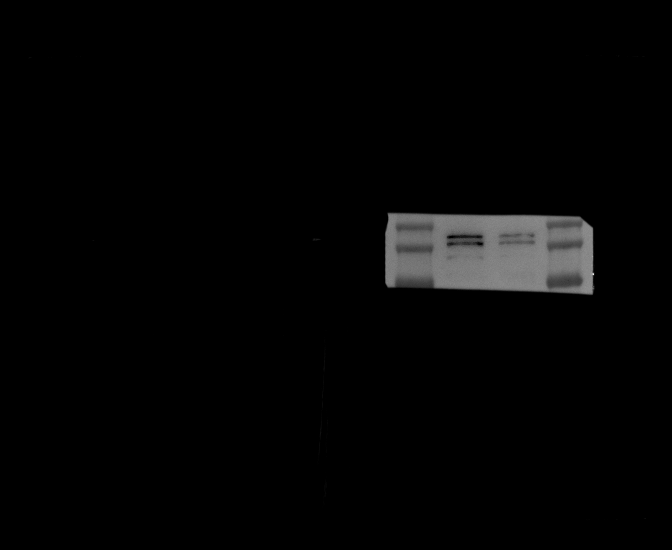

Supplement: Supplementary file 1 [file DataSheet1.zip › original wb/2-DEPDC1.tif]

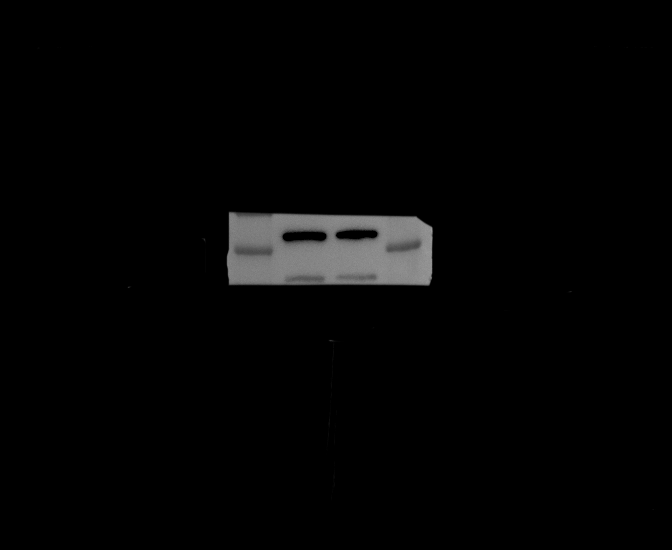

Supplement: Supplementary file 1 [file DataSheet1.zip › original wb/2-GAPDH.tif]

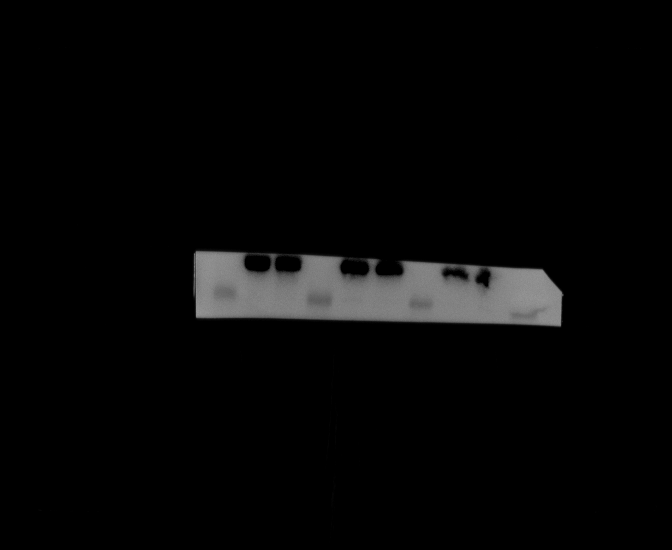

Supplement: Supplementary file 1 [file DataSheet1.zip › original wb/Fig-6B-GAPDH.tif]

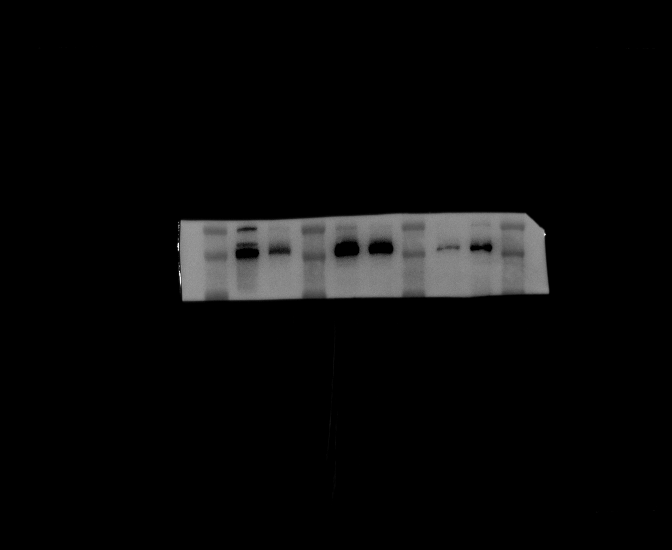

Supplement: Supplementary file 1 [file DataSheet1.zip › original wb/Fig-6B-KIF20A .tif]

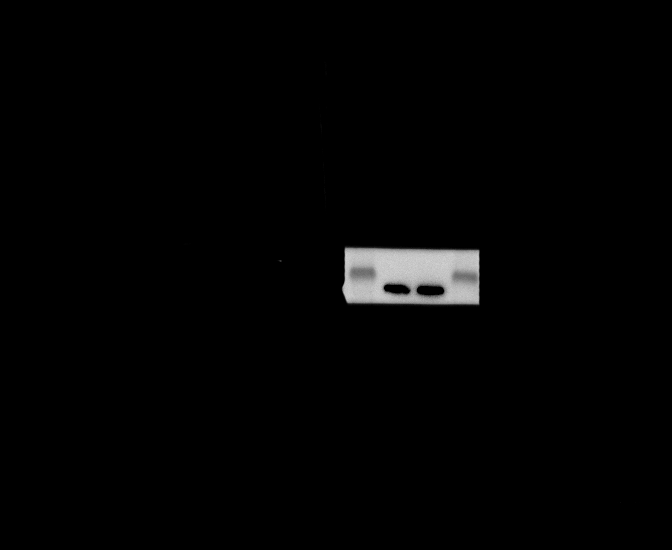

Supplement: Supplementary file 1 [file DataSheet1.zip › original wb/Fig-7A-AKT-2.tif]

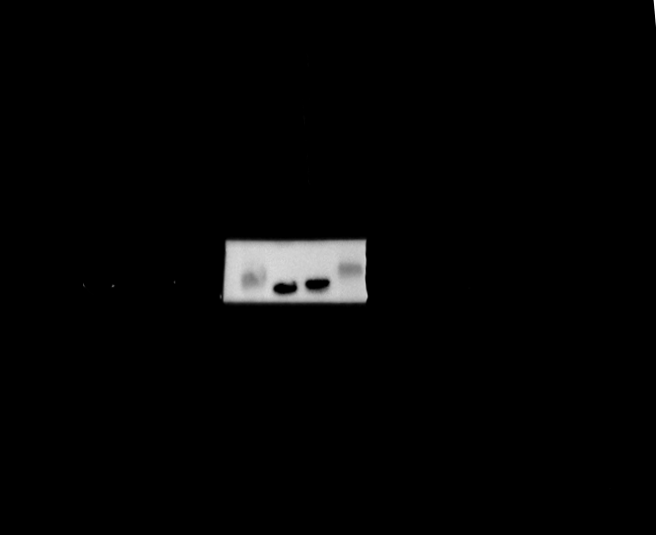

Supplement: Supplementary file 1 [file DataSheet1.zip › original wb/Fig-7A-AKT.tif]

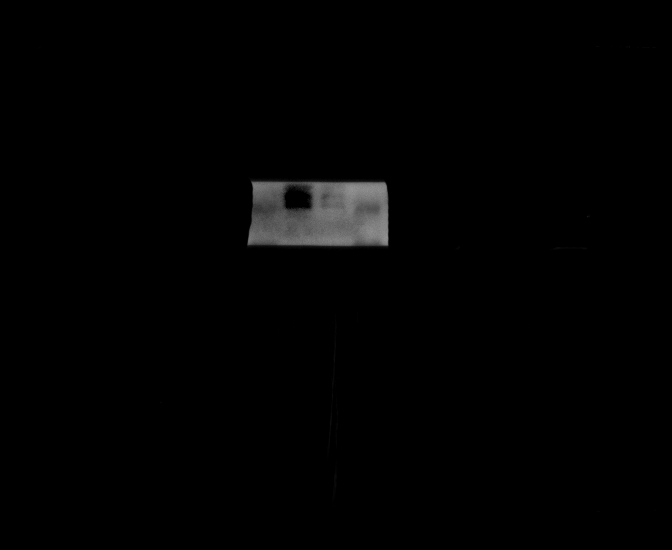

Supplement: Supplementary file 1 [file DataSheet1.zip › original wb/Fig-7A-DEPDC1-2.tif]

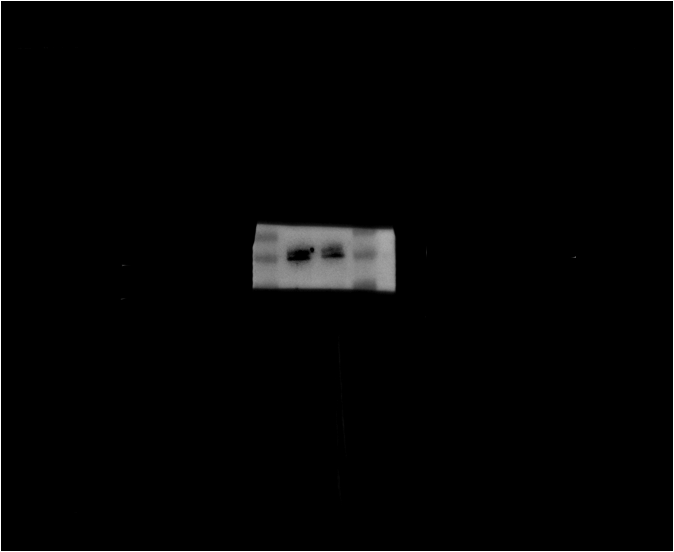

Supplement: Supplementary file 1 [file DataSheet1.zip › original wb/Fig-7A-DEPDC1.tif]

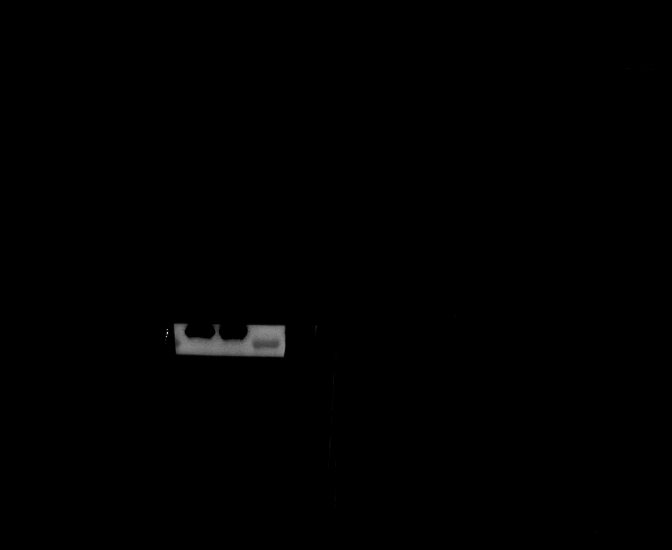

Supplement: Supplementary file 1 [file DataSheet1.zip › original wb/Fig-7A-GAPDH-1.tif]

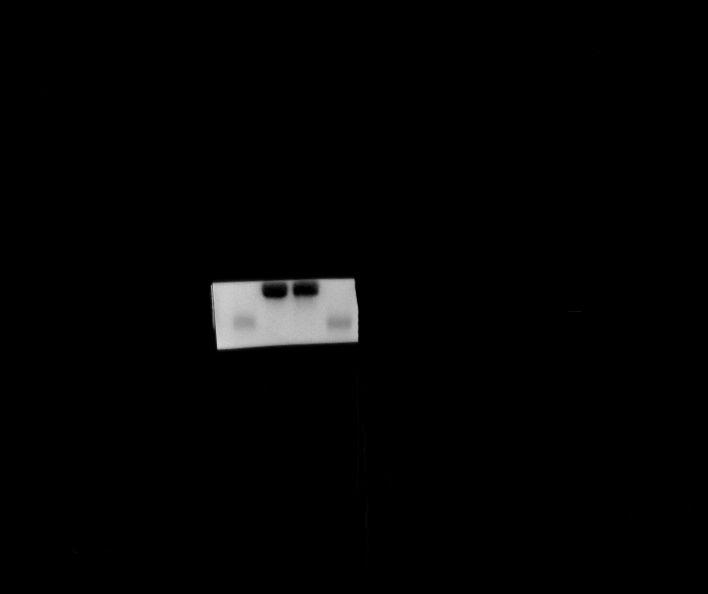

Supplement: Supplementary file 1 [file DataSheet1.zip › original wb/Fig-7A-GAPDH-2.tif]

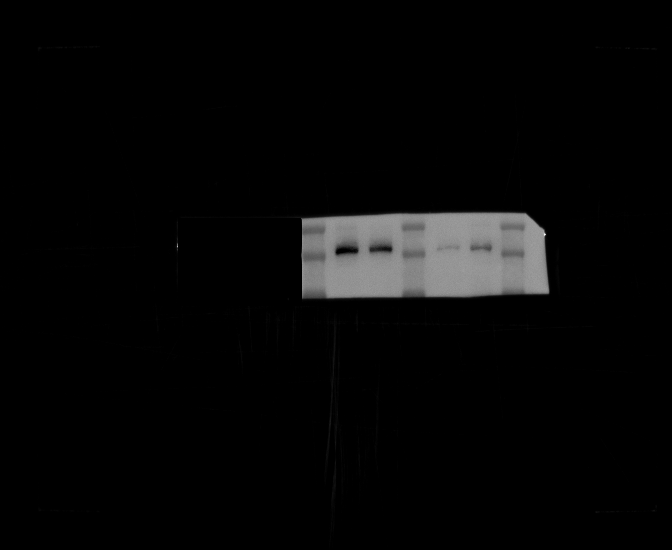

Supplement: Supplementary file 1 [file DataSheet1.zip › original wb/Fig-7A-KIF20A.tif]

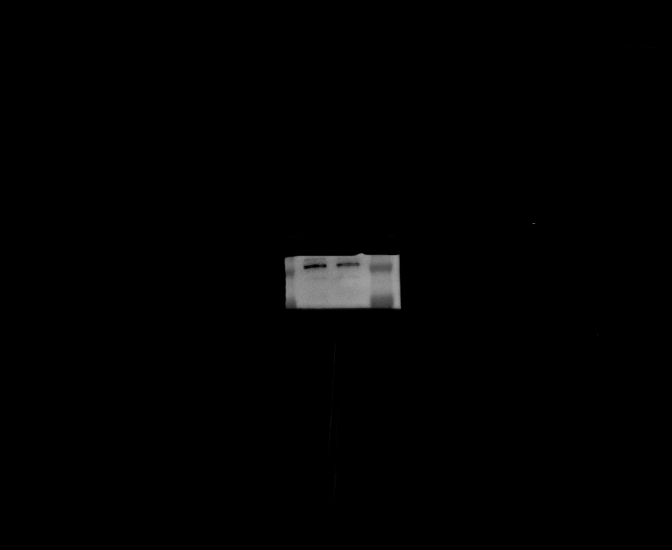

Supplement: Supplementary file 1 [file DataSheet1.zip › original wb/Fig-7A-PI3K-2.tif]

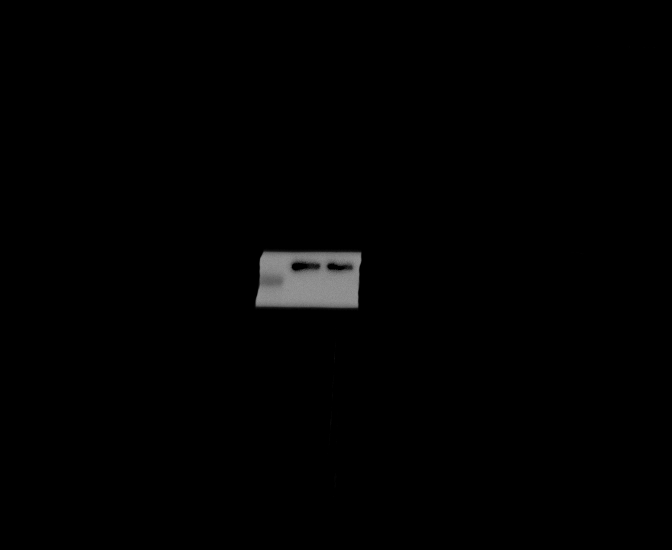

Supplement: Supplementary file 1 [file DataSheet1.zip › original wb/Fig-7A-PI3K.tif]

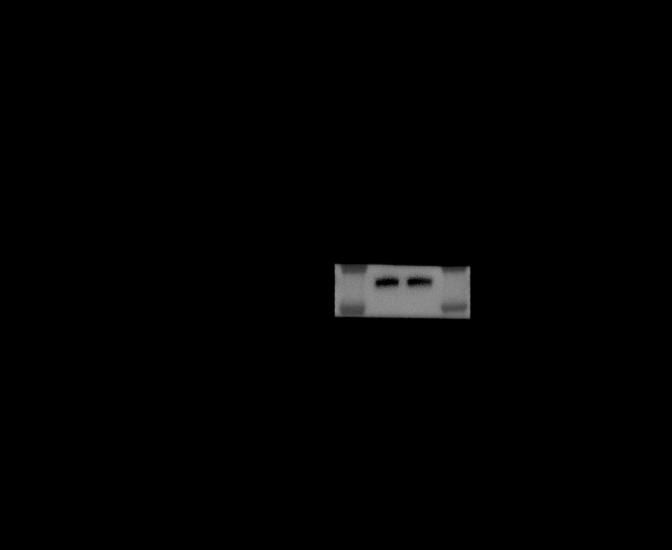

Supplement: Supplementary file 1 [file DataSheet1.zip › original wb/Fig-7A-mTOR-2.tif]

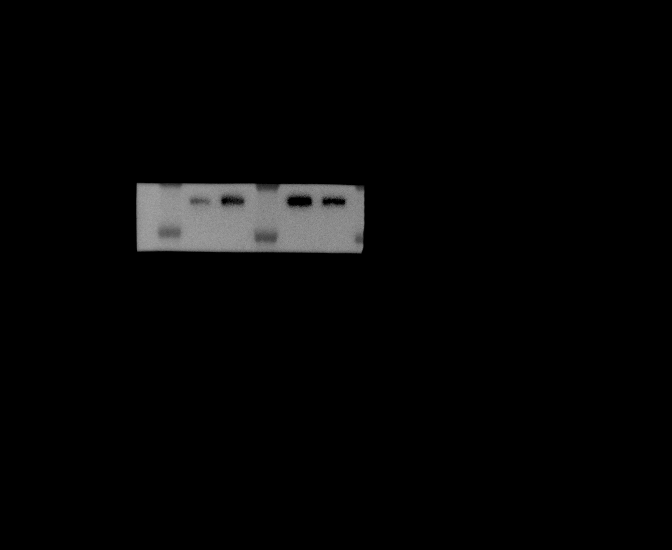

Supplement: Supplementary file 1 [file DataSheet1.zip › original wb/Fig-7A-p-AKT.tif]

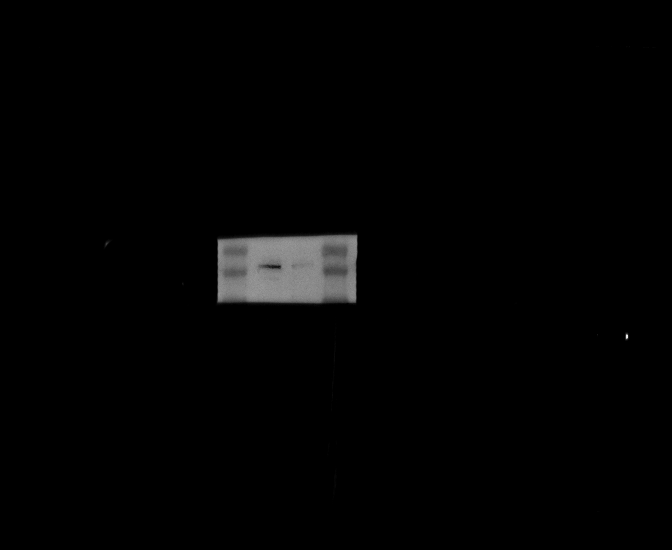

Supplement: Supplementary file 1 [file DataSheet1.zip › original wb/Fig-7A-p-PI3K-2.tif]

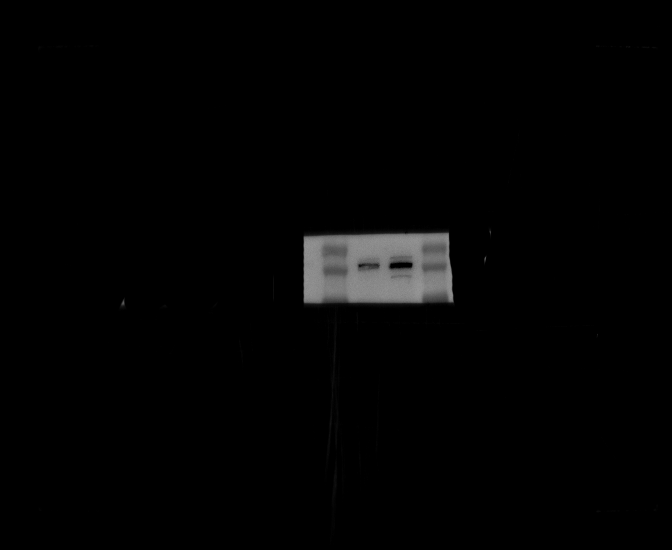

Supplement: Supplementary file 1 [file DataSheet1.zip › original wb/Fig-7A-p-PI3K.tif]

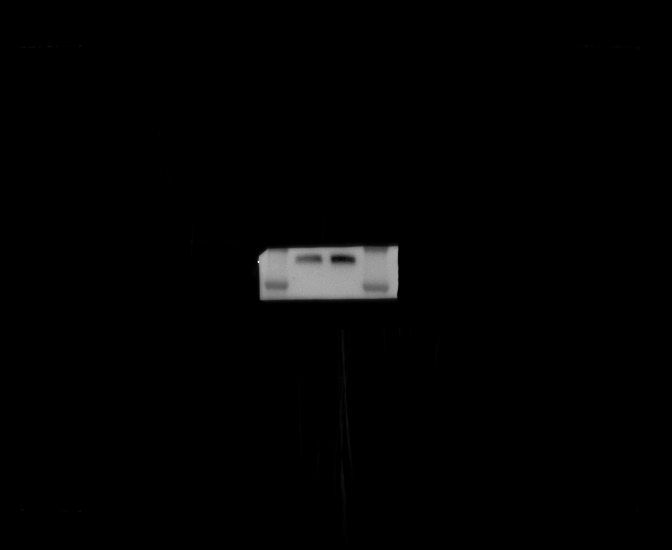

Supplement: Supplementary file 1 [file DataSheet1.zip › original wb/Fig-7A-p-mTOR-1.tif]

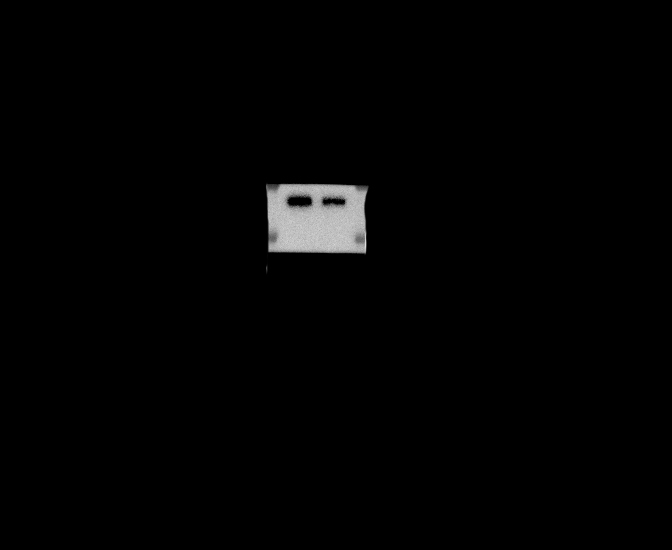

Supplement: Supplementary file 1 [file DataSheet1.zip › original wb/Fig-7A-p-mTOR-2.tif]

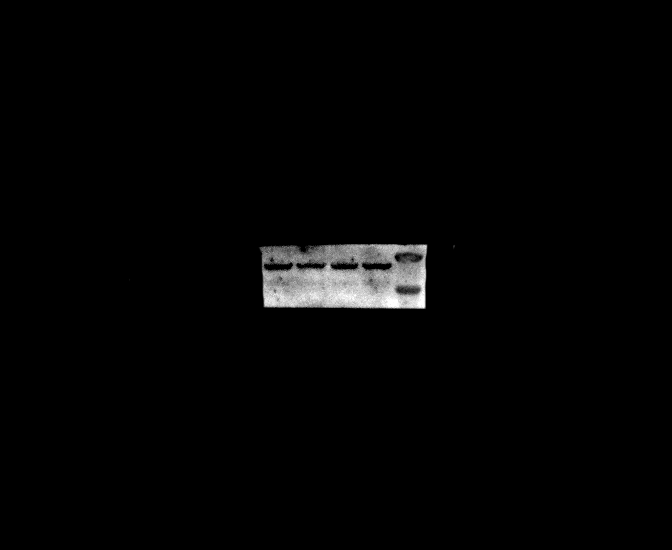

Supplement: Supplementary file 1 [file DataSheet1.zip › original wb/Fig-7B-AKT.tif]

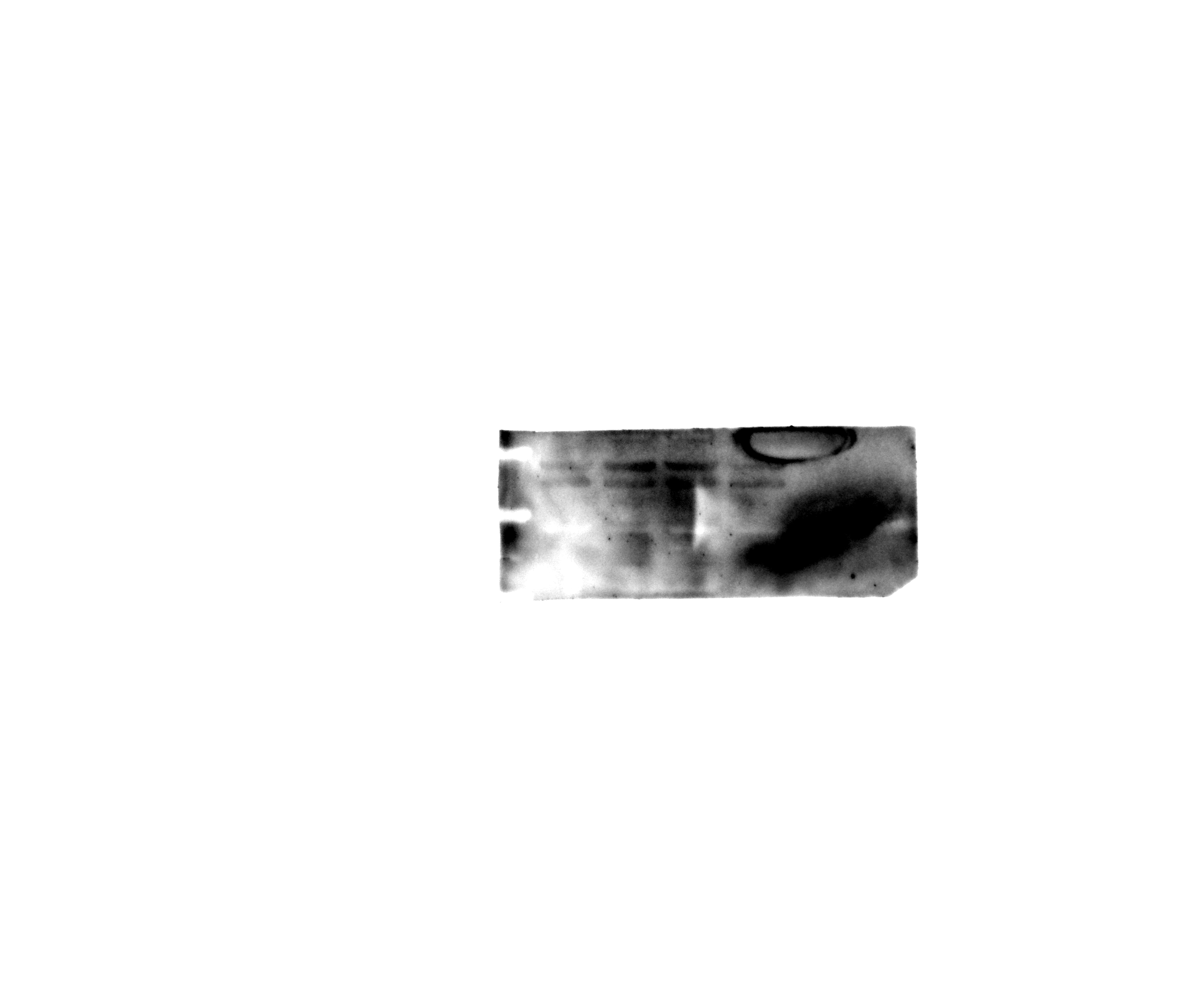

Supplement: Supplementary file 1 [file DataSheet1.zip › original wb/Fig-7B-DEPDC1.tif]

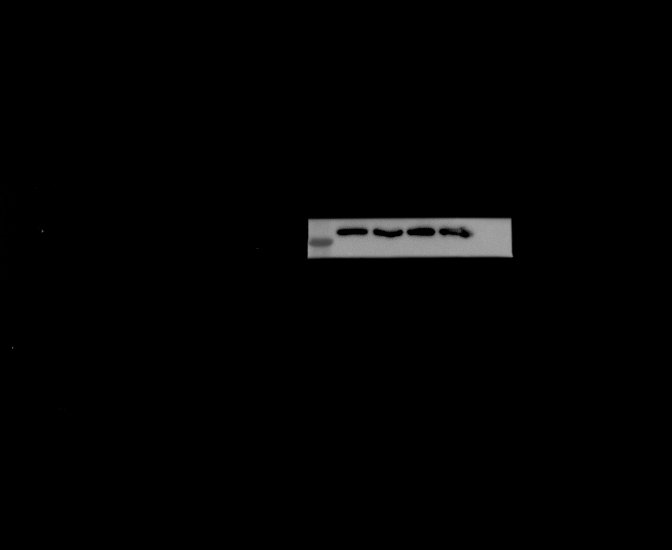

Supplement: Supplementary file 1 [file DataSheet1.zip › original wb/Fig-7B-GAPDH.tif]

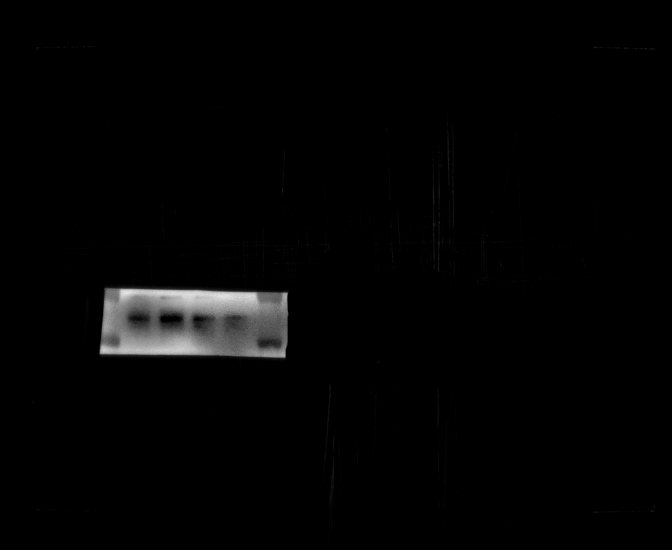

Supplement: Supplementary file 1 [file DataSheet1.zip › original wb/Fig-7B-KIF20A.tif]

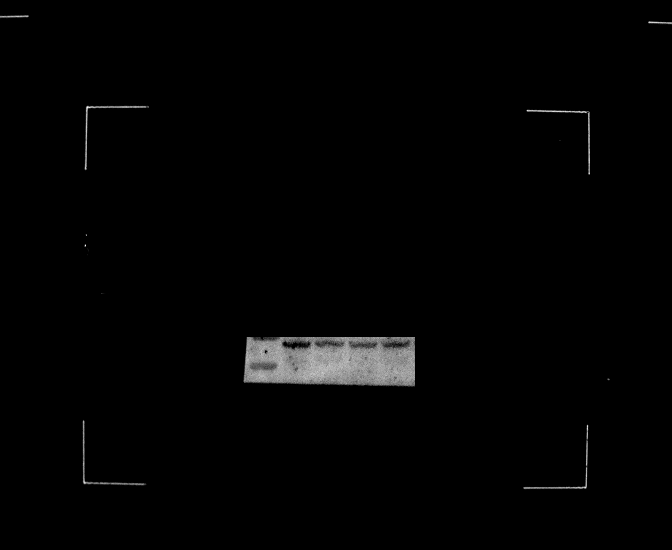

Supplement: Supplementary file 1 [file DataSheet1.zip › original wb/Fig-7B-PI3K.tif]

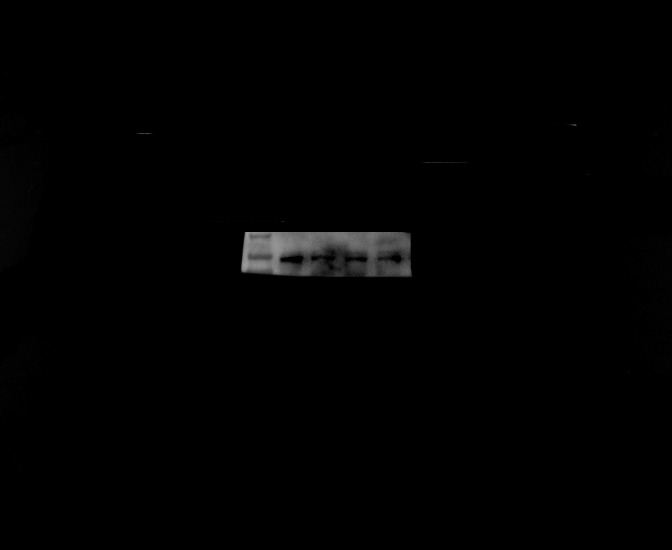

Supplement: Supplementary file 1 [file DataSheet1.zip › original wb/Fig-7B-mTOR.tif]

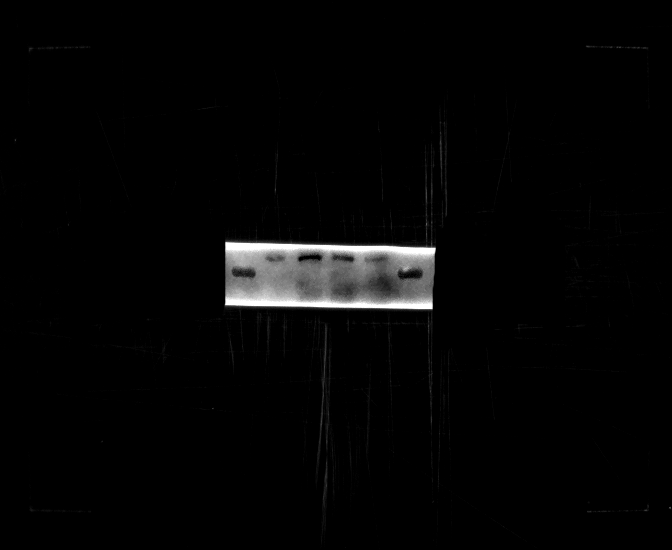

Supplement: Supplementary file 1 [file DataSheet1.zip › original wb/Fig-7B-p-AKT.tif]

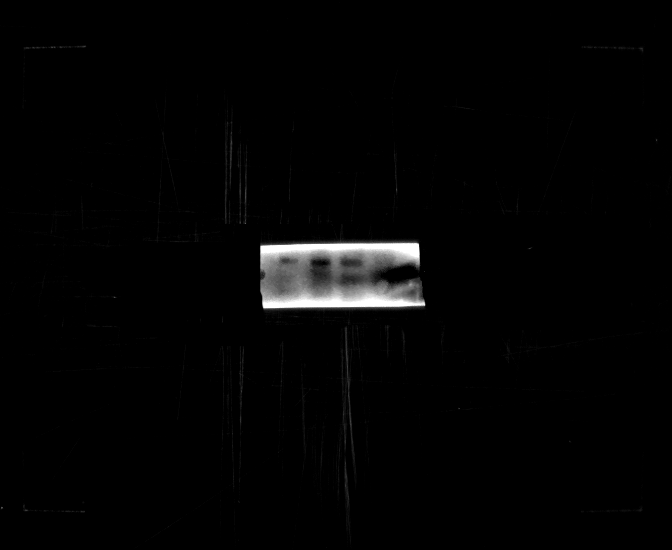

Supplement: Supplementary file 1 [file DataSheet1.zip › original wb/Fig-7B-p-PI3K.tif]

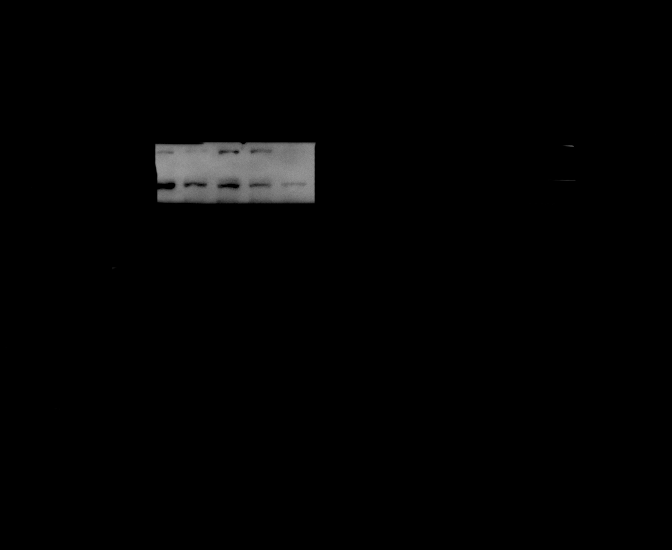

Supplement: Supplementary file 1 [file DataSheet1.zip › original wb/Fig-7B-p-mTOR.tif]

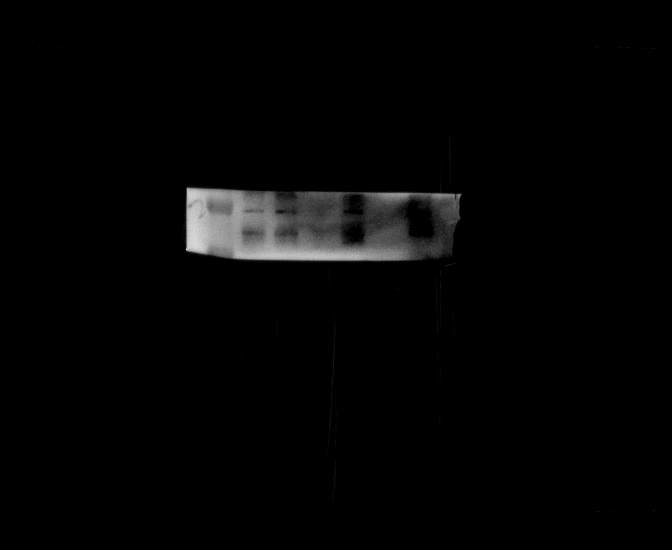

Supplement: Supplementary file 1 [file DataSheet1.zip › original wb/Fig4-DEPDC1.tif]

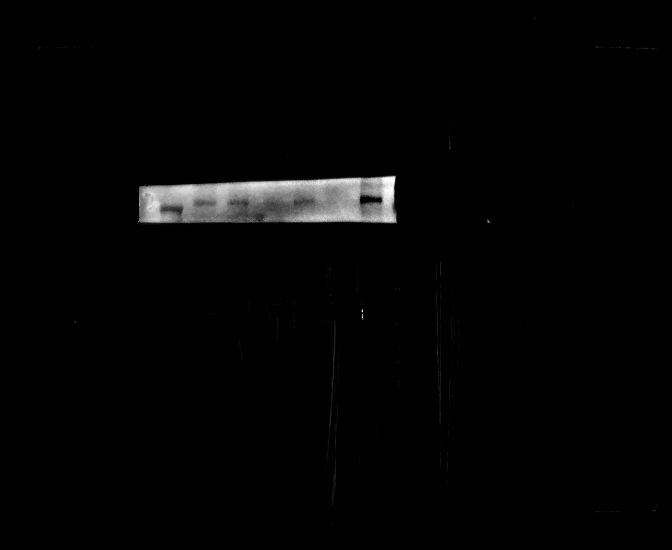

Supplement: Supplementary file 1 [file DataSheet1.zip › original wb/Fig4-KIF20A.tif]

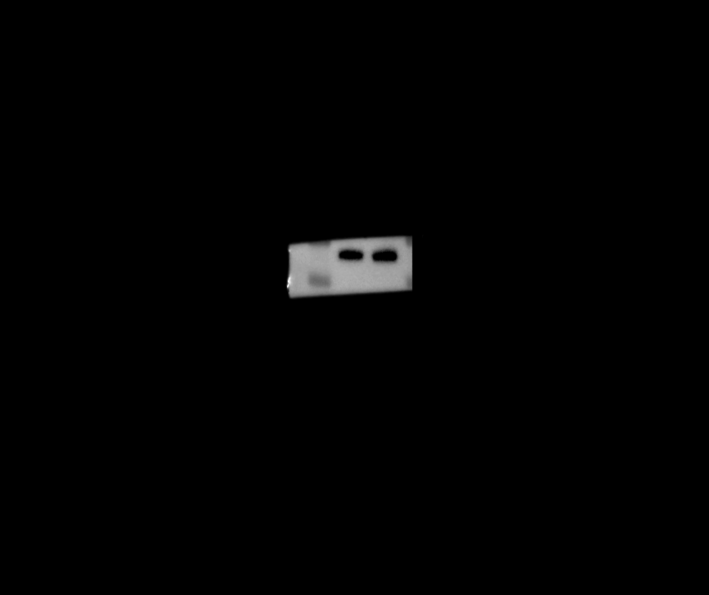

Supplement: Supplementary file 1 [file DataSheet1.zip › original wb/Figure-7A-mTOR-1.tif]

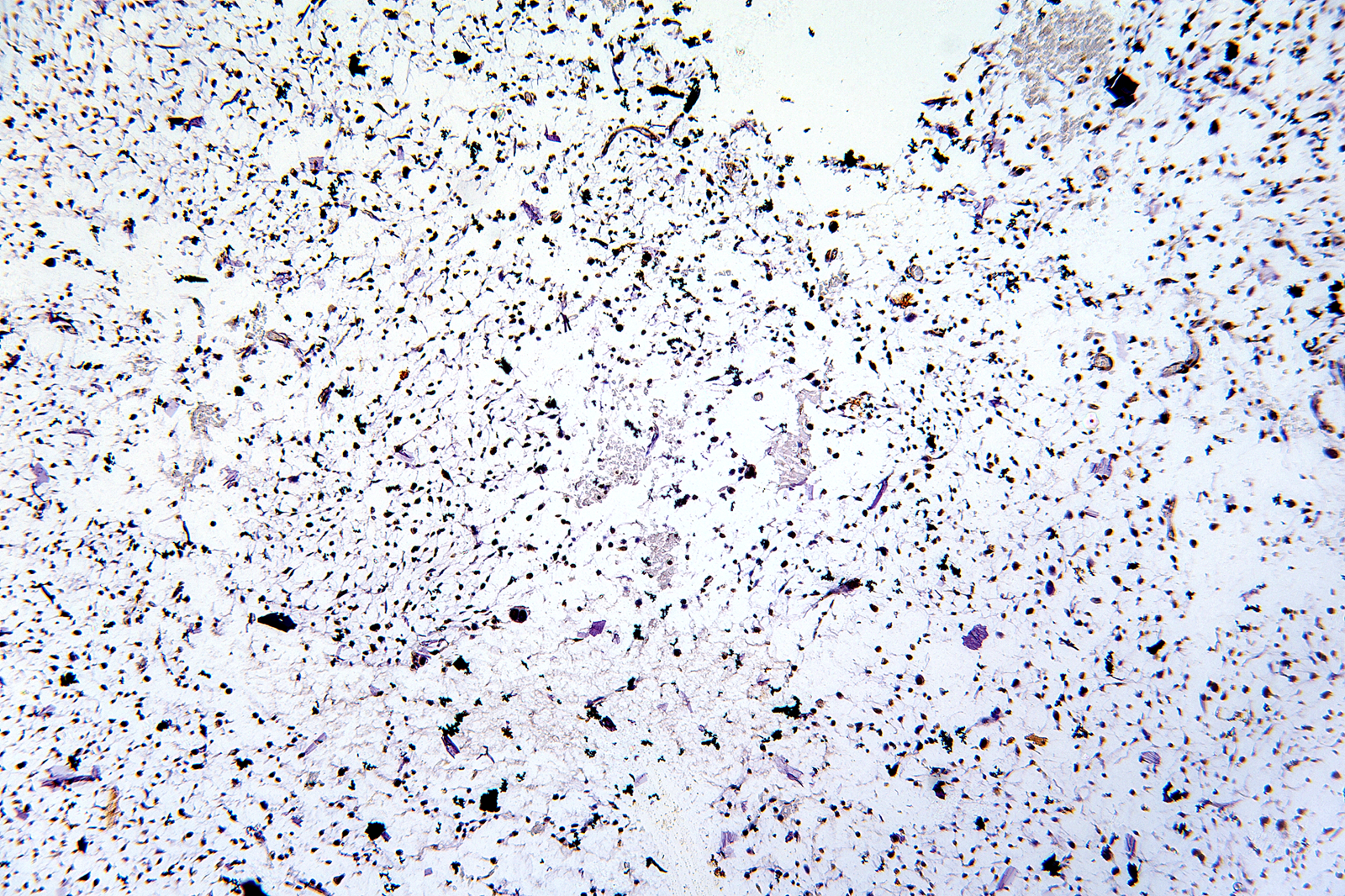

Supplement: Supplementary file 2 [file DataSheet2.zip › IHC-DEPDC1/Fig-2C-DL-10X-1.bmp]

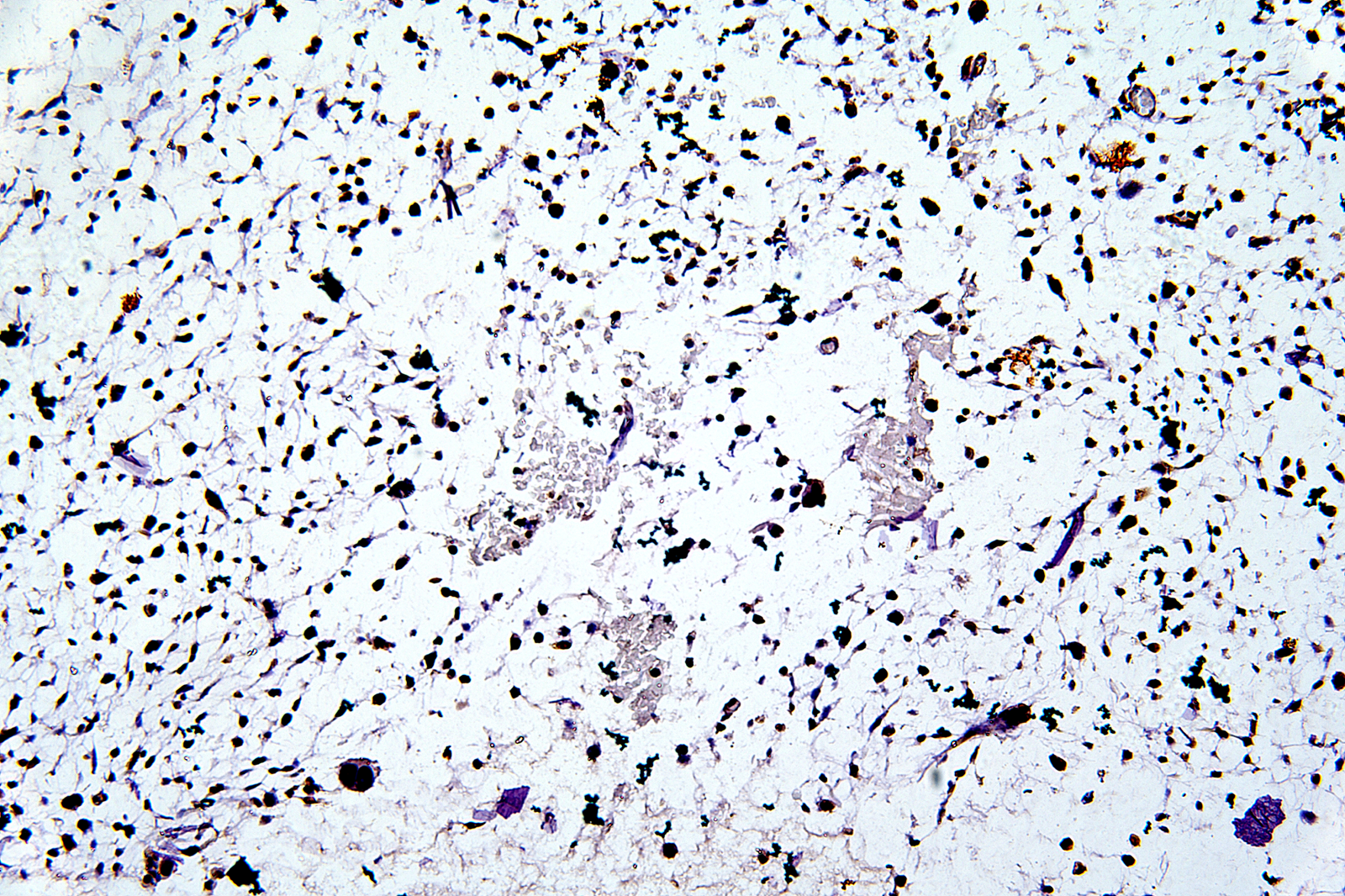

Supplement: Supplementary file 2 [file DataSheet2.zip › IHC-DEPDC1/Fig-2C-DL-20X-1.bmp]

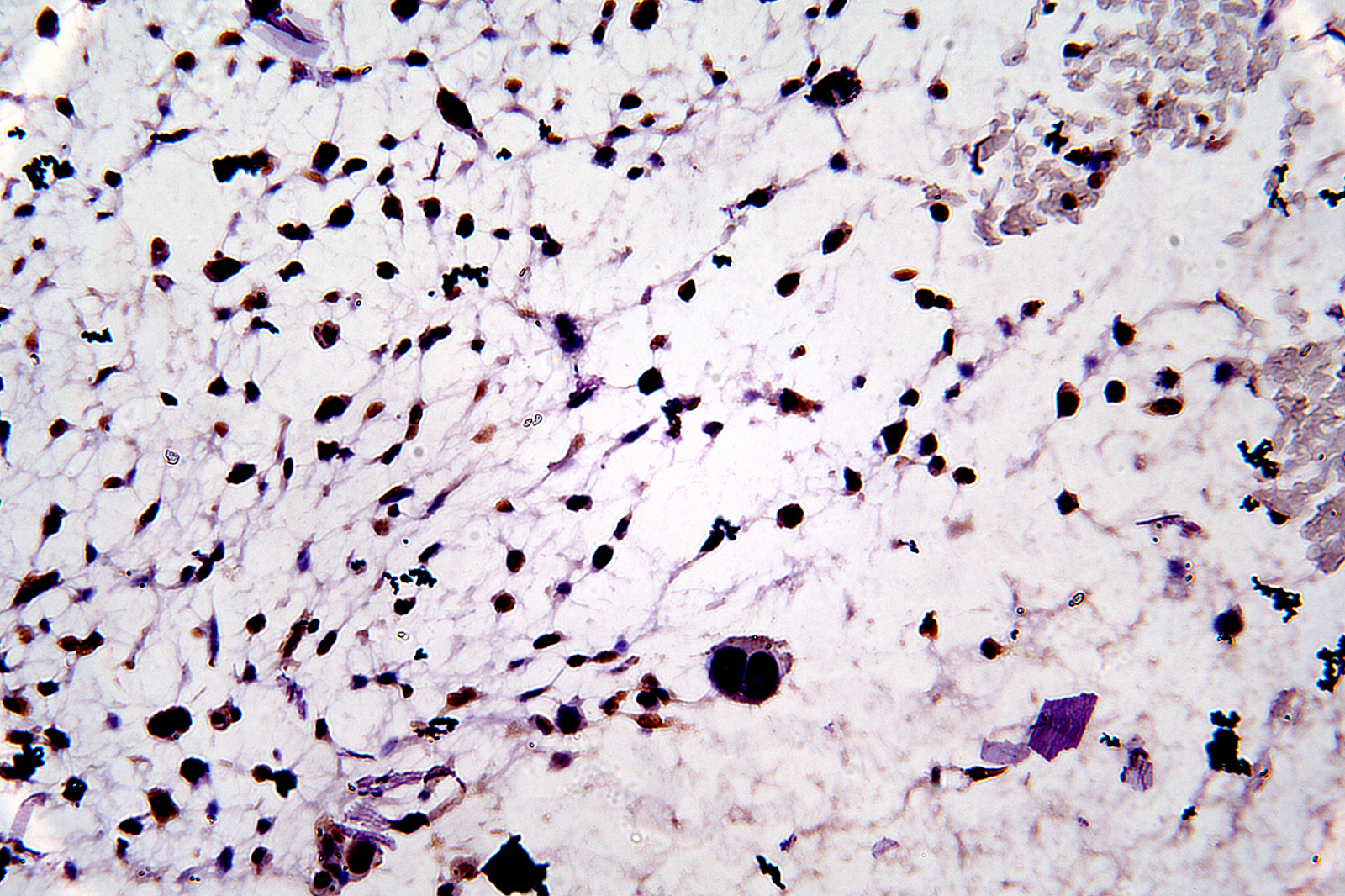

Supplement: Supplementary file 2 [file DataSheet2.zip › IHC-DEPDC1/Fig-2C-DL-40X-1.bmp]

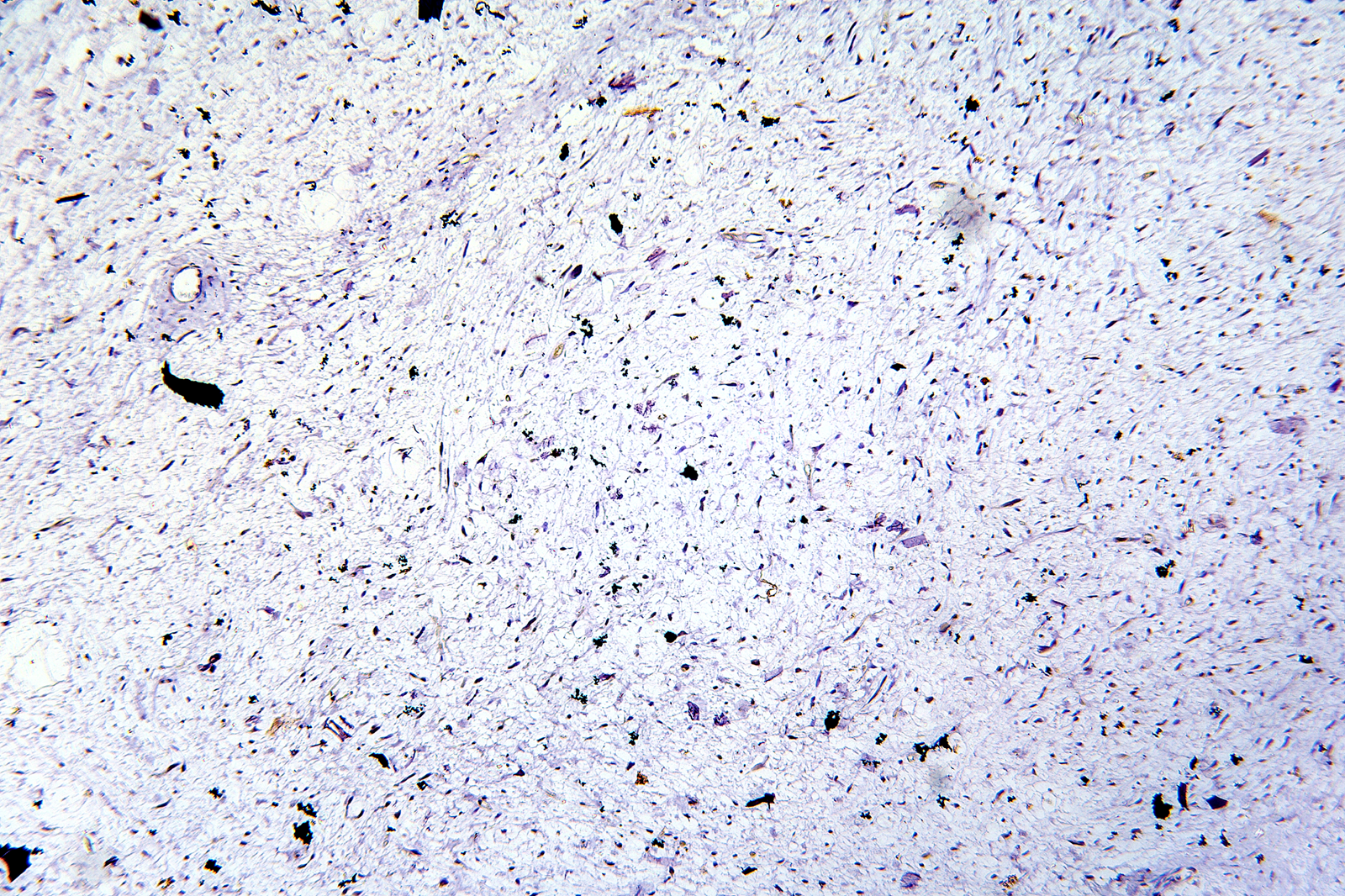

Supplement: Supplementary file 2 [file DataSheet2.zip › IHC-DEPDC1/Fig-2C-WDL-10X-2.bmp]

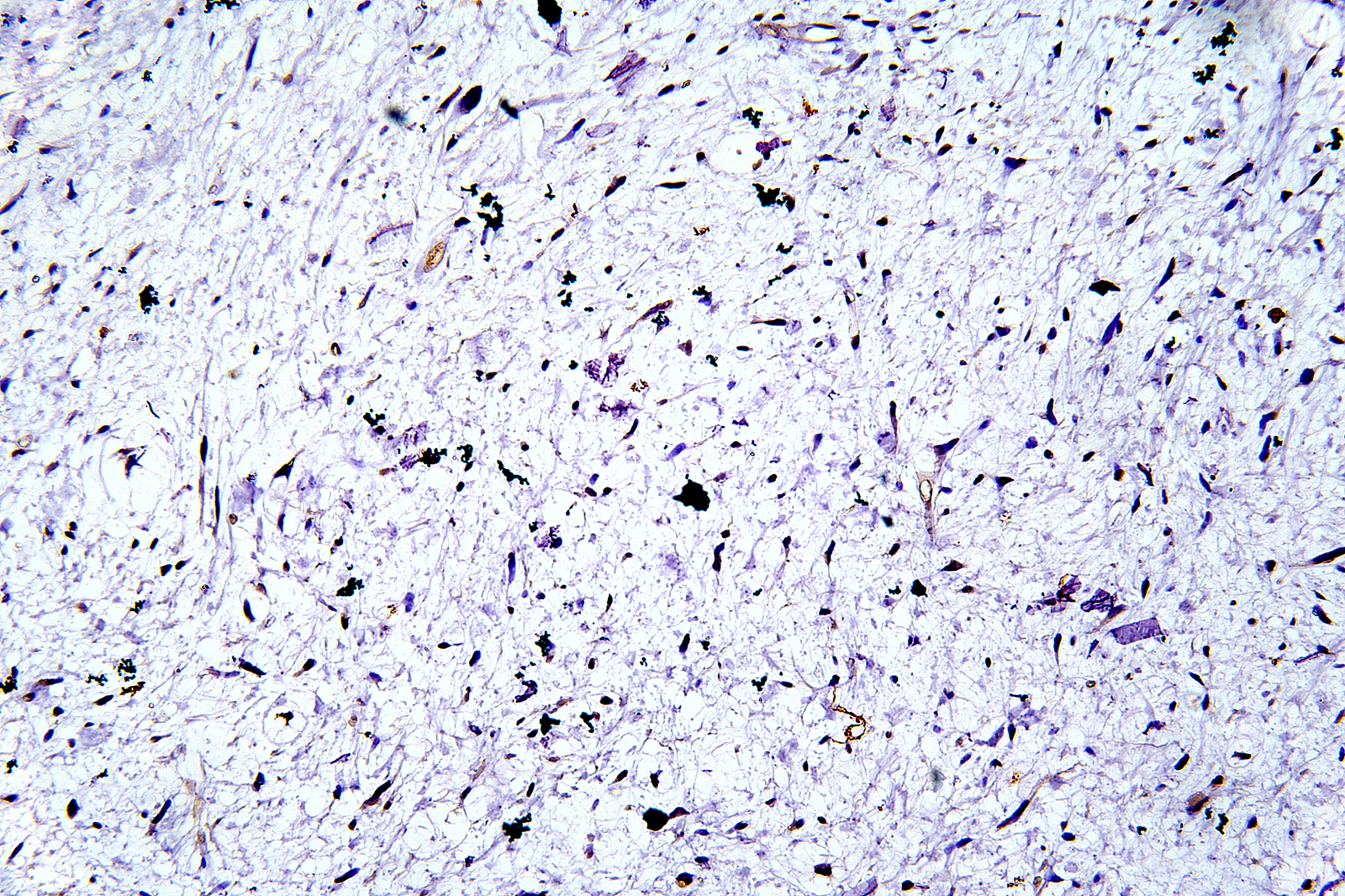

Supplement: Supplementary file 2 [file DataSheet2.zip › IHC-DEPDC1/Fig-2C-WDL-20X-2.bmp]

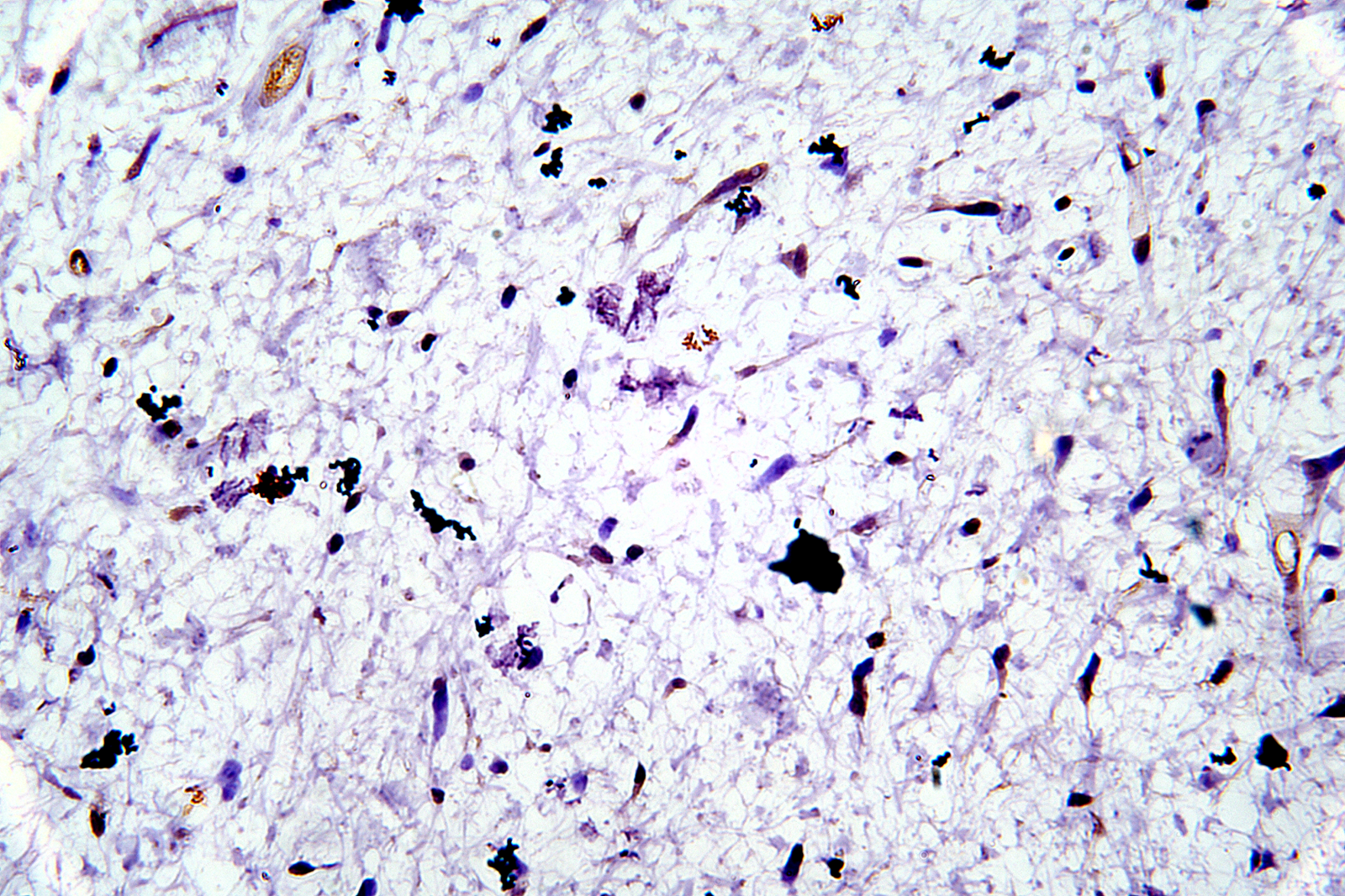

Supplement: Supplementary file 2 [file DataSheet2.zip › IHC-DEPDC1/Fig-2C-WDL-40X-2.bmp]
